# Diagnostic value of microRNAs in asbestos exposure and malignant mesothelioma: systematic review and qualitative meta-analysis

**DOI:** 10.18632/oncotarget.9686

**Published:** 2016-06-01

**Authors:** Luigina Micolucci, Most Mauluda Akhtar, Fabiola Olivieri, Maria Rita Rippo, Antonio Domenico Procopio

**Affiliations:** ^1^ Computational Pathology Unit, Department of Clinical and Molecular Sciences, Università Politecnica delle Marche, Ancona, Italy; ^2^ Laboratory of Experimental Pathology, Department of Clinical and Molecular Sciences, Università Politecnica delle Marche, Ancona, Italy; ^3^ Center of Clinical Pathology and Innovative Therapy, Italian National Research Center on Aging (INRCA-IRCCS), Ancona, Italy

**Keywords:** asbestos, mesothelioma, microRNA, biomarker, systematic review

## Abstract

**Background:**

Asbestos is a harmful and exceptionally persistent natural material. Malignant mesothelioma (MM), an asbestos-related disease, is an insidious, lethal cancer that is poorly responsive to current treatments. Minimally invasive, specific, and sensitive biomarkers providing early and effective diagnosis in high-risk patients are urgently needed. MicroRNAs (miRNAs, miRs) are endogenous, non-coding, small RNAs with established diagnostic value in cancer and pollution exposure. A systematic review and a qualitative meta-analysis were conducted to identify high-confidence miRNAs that can serve as biomarkers of asbestos exposure and MM.

**Methods:**

The major biomedical databases were systematically searched for miRNA expression signatures related to asbestos exposure and MM. The qualitative meta-analysis applied a novel vote-counting method that takes into account multiple parameters. The most significant miRNAs thus identified were then subjected to functional and bioinformatic analysis to assess their biomarker potential.

**Results:**

A pool of deregulated circulating and tissue miRNAs with biomarker potential for MM was identified and designated as “mesomiRs” (MM-associated miRNAs). Comparison of data from asbestos-exposed and MM subjects found that the most promising candidates for a multimarker signature were circulating miR-126-3p, miR-103a-3p, and miR-625-3p in combination with mesothelin. The most consistently described tissue miRNAs, miR-16-5p, miR-126-3p, miR-143-3p, miR-145-5p, miR-192-5p, miR-193a-3p, miR-200b-3p, miR-203a-3p, and miR-652-3p, were also found to provide a diagnostic signature and should be further investigated as possible therapeutic targets.

**Conclusion:**

The qualitative meta-analysis and functional investigation confirmed the early diagnostic value of two miRNA signatures for MM. Large-scale, standardized validation studies are needed to assess their clinical relevance, so as to move from the workbench to the clinic.

## INTRODUCTION

“Asbestos” is a generic term for a group of natural crystalline silicates that have been classified by the International Agency for Research on Cancer (IARC) as “carcinogenic to humans (Group 1)” [1].

These minerals were inexpensive and have been used in extraordinary amounts for decades in a variety of small- and large-scale industrial applications all over the world [2, 3]. Occupational exposure has long been considered the form involving the highest risk of developing asbestos-related diseases (ARDs). Their slow and insidious onset and progression involve that the ban on asbestos production and use and the adoption of asbestos abatement programs over recent decades have not yet eliminated the risk of developing ARDs [3, 4]. In addition, because there is no safe level of exposure, even merely living near plants using asbestos involves a high risk [3, 5, 6]. Repair, renovation, and demolition of asbestos-containing buildings also pose a threat through soil contamination, environmental pollution, and bystander exposure [6, 7]. Asbestos workers themselves can contaminate their homes and cars, exposing family members and housemates [3]. Airborne fibers and leisure activities such as running, horseback riding, and use of all-terrain vehicles that stir polluted soil are further sources [5, 7]. Although current regulations regard only six commercial mineral fibers (actinolite, amosite, anthophyllite, chrysotile, crocidolite, and tremolite) [8], several other mineral fibers found in the environment, such as erionite (zeolite group), whose carcinogenic activity is even greater than that of chrysotile and crocidolite asbestos, are also involved in ARD development [8, 9].

Asbestos exposure entails inhalation of mineral fibers and their accumulation in the lungs, where they cause a variety of adverse effects that include production of reactive oxygen species (ROS), chromosome damage, disturbance of mitosis, gene mutations, alteration of growth factor signaling, defects in the apoptotic machinery, deregulation of methylation status, chronic inflammation, phagocytosis, and aberrant microRNA (miRNA, miR) expression [7, 10]. In the lung parenchyma, asbestos can cause non-malignant inflammatory diseases such as permanent fibrosis (asbestosis) and accumulation of asbestos bodies, which are composed of fibers coated with iron-containing protein [10, 11]. Fibers engulfed in the parenchyma can cause pleural plaques, *i.e.* asymptomatic focal thickenings that are the hallmark of asbestos exposure [12], and abnormal fluid collections, while fibers trapped between the pleural layers and the wall of the chest cavity induce oxidative stress and chronic inflammation, thus promoting carcinogenesis [10]. Moreover, very recent evidence indicates that asbestos causes the release of High Mobility Group Box Protein-1 (HMGB1), which drives the chronic inflammatory process that leads to fibrosis and carcinogenesis [13]. Neoplastic degeneration includes pleural mesothelioma, peritoneal mesothelioma and, albeit rare, mesothelioma of other mesothelial surfaces and bronchogenic carcinoma [10, 11, 14, 15]. A cumulative exposure of 25 fibers/year has been estimated to double the risk of lung cancer [16]. However, it is difficult to determine the level of asbestos exposure in these terms. Current surveillance programs consider both estimated cumulative asbestos exposure (using job-specific questionnaire forms) and radiographic detection of pleural plaques and/or asbestosis [11, 17, 18].

### Malignant mesothelioma: etiopathogenesis and clinical features

Malignant mesothelioma (MM) is an aggressive, lethal cancer arising from the mesothelial cells of pleural (80-90%), peritoneal (10-15%), and pericardial cavities (< 5%) [19]. Its long latency (≥ 30-60 years) [20] and non-specific symptoms often involve late diagnosis and poor survival [21]. MM is among the few cancers that have been causally related to asbestos, erionite [8, 9], ionizing radiation [7], and Simian Virus 40 (SV40), a DNA monkey virus that appears to be a co-carcinogen with asbestos exposure [22–26]. MM has been relatively uncommon until the second half of the 20^th^ century [20, 27]. Its incidence then began to rise in many industrialized countries [28] and is expected to peak between 2015 and 2025 [21, 29]. However, given the intense and widespread use of asbestos worldwide, the health risk related to exposure may be underestimated [29–31]. Globally, one MM case every four/five is believed to go unreported [32]. A recent assessment has attributed about 25% of all MM cases to occupational exposure, 25% to familial exposure, and 50% to environmental exposure [5]. Occupationally exposed patients of a median age of 74 years are more likely to be men, whereas the case distribution at younger median ages (< 40 years) is similar for both genders, and is probably related to environmental exposure [33, 34]. In the latter subjects, MM, uveal melanoma and other cancers have also been related to genetic predisposing factors such as germline mutations in the gene encoding BRCA1 associated protein-1 (BAP1) [35, 36]. Moreover, a growing body of evidence has been relating MM to urban development in rural areas in Cappadocia, North Dakota, Nevada, and New Caledonia after asbestos and erionite fiber contamination [9, 37].

The definitive of pleural malignancy requires invasion and relies on pleural biopsy, pathology, and immunohistochemistry [27, 38]. The differential diagnosis from benign proliferations and other malignancies is highly complex [38, 39], as is histological characterization into epithelioid (50-60% of cases), sarcomatoid (10-20%), biphasic (25-35%), and other, less common subtypes [40, 41]. MM management is controversial and there is currently no cure for it. Only palliative therapies are available. Morbidity and mortality can be reduced by multimodal therapeutic protocols that involve pleurectomy/decortication and extrapleural pneumonectomy, ideally followed by treatment with antifolate pemetrexed and cisplatin, or adjuvant radiotherapy, which are available at some specialized centers [42]. The success and feasibility of such approaches depend on tumor stage and patient performance status and co-morbidity; however, long-term survival is rare and quality of life poor. Advanced stage, poor differentiation, co-morbidities, advanced age, failure to undergo surgical resection, and male gender are associated with a poorer prognosis [21, 42].

The identification of specific, easy-to-analyze biomarkers would greatly help minimally invasive diagnosis, prognosis, and monitoring of response to therapy. Early, accurate diagnosis is critical and would enable patient-tailored care and management. The search for proteins that may serve as MM biomarkers has been ongoing for more than 20 years [43]. Two highly specific proteins, osteopontin and soluble mesothelin-related protein, have been found to lack sensitivity when used individually [44–46], but could be harnessed in multimarker diagnostic panels [47]. According to recent findings miRNAs, a class of short, non-coding RNAs, are differentially expressed during the development and progression of several diseases including tumors, suggesting a role for them as clinical cancer biomarkers [28, 48]. However, the search for miRNAs with diagnostic/prognostic relevance for MM has so far been inconclusive.

### Nature and value of miRNAs in clinical practice

MiRNAs interact with target mRNAs in a sequence-specific manner and provide an additional level of post-transcriptional modulation. They play important roles in several physiological and pathological processes such as cell growth, differentiation, proliferation and metabolism, angiogenesis, stress response, tissue remodeling, disease and malignancy [49–54].

High-quality miRNAs are found in tissue, cells, and body fluids, making them practical, non-invasive markers [55, 57]. It has been established that unique miRNA expression profiles are associated with different cancer types [58, 59], and that about 50% of known human miRNA genes are found in genome areas associated with cancer susceptibility [60–62]. Some miRNA signatures have successfully been applied in lung cancer screening, diagnosis, and follow-up [63], and miRNA combinations may also be sensitive to the effects of pollution [64].

Specific carriers ensure stability of cell-free miRNAs. Extracellular vesicles (EV), *e.g.* exosomes and microvesicles, are actively secreted by malignant cells into surrounding body fluids and may play a role as “communication shuttles” [65, 66]. The finding that cell-free miRNAs are also associated with Argonaute (Ago) proteins and are mostly EV-free has suggested that extracellular Ago2-miRNA complexes may be residuals of dead cells [67]. The hypothesis has also been advanced that cells could release a functional miRNA-induced silencing complex into the circulation [68]. In addition, functional targeting abilities have also been demonstrated for high-density lipoproteins (HDLs) that transport endogenous miRNAs to recipient cells [69]. Such carriers are a unique source of specific miRNA biomarkers.

In this study, data on deregulated miRNAs reported in specimens from asbestos-exposed and MM subjects were systematically reviewed, and a qualitative meta-analysis was conducted to assess their diagnostic potential and find evidence-based diagnostic miRNA signatures for asbestos exposure and MM.

## RESULTS: OVERVIEW, ANALYSIS, AND SUMMARY OF MAIN RESULTS

### MiRNAs related to MM: state of the art

The variety of techniques and approaches adopted in the 39 selected studies makes it difficult to classify them and summarize their findings concisely. However, study design enabled their subdivision into 19 papers largely addressing miRNA profiling, 11 performing single-miRNA expression analysis, and 9 evaluating miRNA activity by functional assays. As noted below (5.1, Literature search and screening), the latter articles were considered only for their qualitative contribution. Moreover, only 6 of the 39 studies investigated miRNA-induced epigenetic modifications, and another explored miRNA deregulation in asbestos-related lung cancers, including adenocarcinoma, adenosquamous carcinoma, small cell lung cancer and large cell lung cancer [70]. Since studies of peritoneal and pericardial mesothelioma are not available, in the present review all MM-associated miRNAs refer to pleural mesothelioma.

#### MiRNA profiling studies

Since a multitude of miRNAs function in networks to modulate gene expression pathways, we considered expression profiling as the most effective high-throughput screening approach to analyze hundreds of miRNAs simultaneously.

- *MiRNA profiling in MM tissues*. Guled and colleagues were the first to document the deregulation of a multitude of miRNAs in MM samples both compared with normal tissue (pericardium from healthy subjects) and among the epithelioid, sarcomatoid, and biphasic subtypes [71]. Further studies seeking to distinguish MM from other cancers first identified 7 MM-specific miRNAs, including miR-200b, miR-200c, miR-141 and miR-429, as useful tools for differential diagnosis from adenocarcinoma, but not among different histological types [72]. Differential expression of miR-193-3p, miR-192 and miR-200c has subsequently been demonstrated in MM tissue compared with carcinomas [73], whereas miR-29c* (miR-29c-5p according to the upgraded nomenclature) has been proposed as a prognostic biomarker [74]. Combined analysis of miRNA expression patterns and functional assays has highlighted that miR-1 is down-regulated in MM compared with normal mesothelium, and that its forced expression can inhibit cell proliferation and apoptosis [75]. By a similar approach, miR-145 loss has been seen to distinguish MM from normal mesothelial tissue [76]. Andersen's group has identified four miRNAs as diagnostic classifiers capable of differentiating MM from non-cancer samples with high overall accuracy, and demonstrated that chemotherapy reduces their differential expression [77]. The two most recent studies in the field have disclosed that 19 miRNAs are differentially expressed in MM, chronic pleural inflammation and mesothelial hyperplasia compared with non-cancer/non-inflammatory tissue [78], and that the expression of 6 miRNAs enabled predicting survival in MM patients [79]. Eleven significantly up-regulated miRNAs have been identified in MM compared with benign asbestos-related pleural effusion [80].
- Thirteen novel asbestos-related miRNAs and inversely correlated target genes have been identified by an integrative analysis of miRNA, mRNAs and copy number alterations of chromosomal regions in tissue samples from lung cancer patients with high asbestos exposure and without exposure [70].
- *MiRNA profiling in MM cells.* Use of MM cell lines allows to address the problem of collecting suitable numbers of MM tissue samples. The results of the first two studies, exploring the *in vitro* expression profiles of MM cell lines compared with mesothelial cells [81, 82], were questioned by a paper reporting the opposite behavior of some miRNAs in MM tissue [83]. Ivanov and co-workers suggested that miR-31 could serve as a prognostic factor because its loss *in vitro* had a pro-tumorigenic effect on MM cell lines [84]. The first studies shifting the search for deregulated miRNAs from tissues to the circulation were conducted by Santarelli *et al.* [85] and Tomasetti *et al.* [86]. Whereas the former work examined tissue profiling and validated the clinical significance of miR-126 in sera from MM patients, the latter paper suggested that circulating miR-126 is a sensitive disease marker that should however be used in combination with other biomarkers, such as mesothelin, to increase specificity [86].
- *Profiling of circulating miRNAs in MM patients.* Circulating miRNAs are promising candidates for the development of non-invasive techniques for early cancer detection/diagnosis. A new approach, based on the evidence that tumors generate a characteristic miRNA fingerprint in the cellular fraction of peripheral blood [87], has shown that miR-103 levels were able to discriminate MM patients from asbestos-exposed subjects and healthy controls [88]. Combining miR-103 (miR-103a-3p according to the upgraded nomenclature) with mesothelin has improved diagnostic performance [89]. The first miRNA profiling study in plasma/serum was reported by Kirschner *et al.*, who demonstrated that miR-625-3p levels showed fairly high specificity, accuracy, and sensitivity in differentiating MM from asbestosis patients [90]. Finally, the most recent study has identified two different serum miRNA signatures correlating respectively with MM histological subtype and clinical outcome [91].

#### Single-miRNA expression and functional analyses

Several investigations have addressed individual miRNAs. Fassina and colleagues studied the modulation of the epithelial-mesenchymal transition and found that miR-205 downregulation correlates with the mesenchymal phenotype and with a more aggressive disease [92]. A study of MM cell lines examining the overexpression of the EZH2 gene, which encodes core components of polycomb repressor complex-2 (PRC-2), involved in the pathogenesis of different cancers, found low expression of its mRNA regulators, miR-101 and miR-26 [93]. Analysis of the combined expression of miR-15 and miR-16 documented their significant downregulation and tumor-suppressing function in MM compared with normal mesothelium; moreover, their forced expression appeared to be related to inhibition of proliferation [94]. It has also been reported that miR-23a and miR-27a modulation in MM induce silencing of ZIC1, a potential tumor suppressor gene involved in apoptosis [95]. The PVT1 locus is another oncogene acting as a non-coding RNA through different, alternatively spliced isoforms. Its frequent copy number gain in MM cell lines, combined with miR-1204 depletion at the same locus, promotes overexpression of anti-apoptotic genes and the malignant phenotype [96]. Significant miR-31 downregulation has been reported in MM macro-dissected tissue *vs.* reactive mesothelial proliferations, whereas miR-31 upregulation is significantly associated with a worse prognosis in patients with sarcomatoid MM [97]. Down-regulated miR-223 expression has been reported to modulate STMN1, which has an important role in microtubule dynamics; both are involved in the JNK signaling pathway [98]. Comparison of miR-192, miR-193a-3p, and miR-200 family expression in normal pleura and MM specimens has highlighted a significant reduction in miR-192 and miR-193a-3p in MM tissue. Finally, restoration of miR-193a-3p levels has been reported to inhibit MM cell growth and xenograft tumors *in vivo* [99].

#### MiRNAs and epigenetic modifications

Similar to protein-coding genes, miRNA-encoding genes are also affected by epigenetic changes. DNA methylation status is commonly altered in tumor cells, and significant mesothelioma modulation has been associated with exposure to carcinogens like tobacco, nickel, and asbestos [100]. An aberrant methylation status and silencing of miR-34b and miR-34c has also been described in MM specimens [101]. Extensive functional investigation of miR-34b/c activity has suggested that downregulation of miR-34 family members induces proliferation and invasion of human mesothelial cells, thus playing an important role in carcinogenesis [102]. Preclinical evaluation of adenovirus-mediated miR-34b/c gene therapy has shown promise in the treatment of malignant pleural mesothelioma (MPM) [103]. The therapeutic approach based on miR-34 family members promotes radiation-induced apoptosis [104] also in epithelial MM cells [105]. A digital real-time methylation-specific PCR assay, developed by Muraoka's group to quantify miR-34b/c methylation in serum-circulating DNA, suggests the association of this family with MPM; the approach could be the basis for a new detection system [106].

#### Functional studies without expression analyses

Studies that do not provide miRNA quantification may nonetheless have potential translational importance, and were included in the present review, to provide a more exhaustive picture. Treatment of MM cell lines with the chemotherapeutic agent ranpirnase (Onconase^(R)^) induced miR-17* upregulation and miR-30c downregulation; this indicates that these miRNAs have anti-tumor activity, as also confirmed by downregulation of NF-kB and downstream targets [107, 108]. Let-7 family expression appears to be induced by activation of EphrinA1, the ligand of Ephrin type-A receptor, which suppresses MM cell growth by targeting the RAS proto-oncogene [109]. A recent paper has confirmed the hypothesis that miR-126 affects mitochondrial energy metabolism, and that its upregulation *via* oxidative stress induces MM tumor suppression [110]. Finally, assessment of miRNA expression profiles in a panel of drug-sensitive and drug-resistant MM cell lines, to explore new therapeutic options, has recently suggested a correlation between the miR-379/411 cluster and drug resistance. MiR-379 and miR-411 have been seen to promote invasion and drug resistance by direct targeting of IL-18 in MPM cell lines [111].

### Role of SV40 miRNAs in MM

The first demonstration of a connection between human MM and SV40 was the discovery of SV40-like DNA sequences in MM specimens, though not in matching lung samples, from individuals contaminated exclusively by asbestos [112]. Asbestos and SV40 can act independently or as cofactors in tumor development [24, 112]. Similarly, expression of the SV40 large-T antigen has been demonstrated in mesothelioma, but not in surrounding lung parenchyma [112]. SV40 large-T antigen complexes with p53 and leads to activation of insulin-like growth factor-I promoter and eventually to stimulation of malignant mesothelial cell growth [113–115].

**Figure 1:**
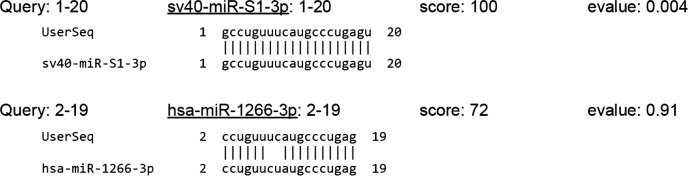
Alignment of sv40-miR-S1-3p to human miR-1266-3p Search parameters: Sequence ID: MIMAT0003345 (sv40-miR-S1-3p), MIMAT0026742 (has-miR-1266-3p); search algorithm: BLASTN; sequence database: mature; Evalue cutoff: 10; max alignments: 100; word size: 4; match score: +5; mismatch penalty: 4.

SV40 encodes a long antisense RNA, the miR-S1 stem-loop, which leads to production of two miRNAs, sv40-miR-S1-5p and sv40-miR-S1-3p. In late infection stages, these miRNAs target and cleave early viral mRNA, reducing the expression of SV40-T antigens without reducing the production of infectious virus [116]. Cells infected with wild-type SV40 virus, unlike those infected with a mutant SV40 virus lacking miRNAs, are less sensitive to lysis by cytotoxic T cells, and because they exploit the miRNA pathway [117]. Polyomavirus strains with severely attenuated miRNA expression arise infrequently *in vivo*, and loss of viral miRNAs can occur in conditions of immune suppression [118]. Significantly, such autoregulation of gene expression is conserved in several polyomaviruses, and it is conceivable that viral miRNAs may target multiple host genes besides own transcripts. This would be even likelier if viral miRNAs had similar sequences to host miRNAs. We examined this possibility by comparing SV40-encoded miRNAs to the entire database of human miRNAs using sequence alignment. Surprisingly, sv40-miR-S1-3p showed a high level of similarity to human miR-1266-3p, as shown in Figure 1. Deregulation of mir-1266 gene family miRNAs has been related to recurrence and metastasis in patients with estrogen receptor-positive breast cancer [119], gastric cancer growth and invasion [120], and psoriasis vulgaris [121].

A RT-PCR study of SV40-encoded miRNAs in MM tissue specimens failed to detect viral miRNAs [122]. The authors reported that SV40 miRNAs do not contribute to MM tumorigenesis; however, the patients' unknown SV40 virus infection status does not allow to draw this conclusion. Indeed, the presence and expression of SV40 in MM [123] and the absence of SV40-like DNA sequences in patients not exposed to the virus [124] have been reported by several groups. SV40 may actually contribute to the development of those MM forms that are not due to asbestos exposure, and facilitate asbestos-mediated carcinogenicity [125].

### Identification of miRNA signatures in MM and asbestos exposure

This brief overview documents that a multitude of miRNAs are differentially expressed in specimens from MM, asbestos-exposed, and healthy subjects.

Whereas identifying signatures with clinical relevance requires experimental validation, individual asbestos-related and MM-related miRNAs are likely to be consistently reported in different papers. A logical approach to distinguishing relevant from spurious miRNAs is therefore to focus on those described more frequently. Accordingly, the traditional vote-counting method (see under 5.3. Vote-counting methods) was applied to all the miRNAs that have been reported to be deregulated in the 30 studies selected by our literature search. We found that the most frequently reported miRNAs had been described in 5 and 4 papers, and listed them in Table 1; all the 213 identified including the others, which had been reported in 3, 2, or 1 study, are listed in Supplementary Table 1. In the two tables, up- and down-regulated miRNAs are marked by arrows pointing up and down, whereas deregulated miRNAs, *i.e.* those for which no consistent direction has been described, those found to be deregulated in different MM histotypes, and those for which the relevant paper provided insufficient data, are accompanied by a horizontal arrow. MiRNAs could not be ranked by the number of samples in which they had been found or by average fold changes, because most papers did not provide this information.

**Table 1:** Most frequently reported miRNAs in malignant mesothelioma and asbestos exposure evaluated by a traditional vote-counting method

Comparison status among groups	miRNAs	MM *vs*. normal tissue / benign proliferations		MM *vs*. other cancers or different histotypes		MM blood samples *vs*. normal blood samples		MM cells *vs*. normal cells	
**MiRNAs deregulated in the four comparison categories**	miR-17-5p	↓	[83]	→	[82], [79]	↓	[88]	↑	[81], [82]
	miR-20a / miR-20a-5p	↑	[80]	→	[79]	↓	[88]	↑	[81], [82]
	miR-21 / miR-21-5p	↑	[77]	→	[82], [79]	↓	[88]	→	[82]
	miR-29c* / miR-29c-5p	↓	[90]	→	[74], [79]	↑	[90]	↓	[74]
	miR-30c	↓	[85]	→	[82]	↓	[88]	↑	[82]
	miR-92 / miR-92a / miR-92a-3p	→	[90]	→	[79]	↑	[90]	↑	[81]
		↑	[80]			↓	[88]		

Note: deregulated miRNAs extracted from relevant papers and classified based on four comparison categories: a) MM tissue *vs.* normal or non-cancer tissue; b) MM tissue *vs.* other cancer tissues; c) MM blood samples *vs.* normal blood samples; and d) MM cell lines *vs.* normal cell lines. MiRNAs are reported as being up-regulated, down-regulated or deregulated based on the relevant studies.

↑: up-regulated, ↓: down-regulated, →: deregulated miRNAs. For the latter clear expression information is not provided in the corresponding article, or they have been found to be deregulated in different MM histotypes. Numbers to the right of each arrow are the references numbered according to the reference list. The new nomenclature is reported where the old miRNA name could be ambiguous. Because of space limitations, the full table and details of miRNA behaviors are reported in Supplementary Table 1.

In most cases, miRNAs from the same sample type shared the same direction of deregulation; notably, miR-101-3p, miR-15b-5p, miR-16-5p, miR-192, and miR-195-5p were consistently found to be down-regulated in all sample types. Other miRNAs exhibited an inconsistent expression, possibly in relation to different types of assays, storage methods, or biopsy collection; for example, laser capture micro-dissected (LCM) tissue may show a different miRNA deregulation profile compared with a conventional biopsy, due to less interference from surrounding normal tissue. Moreover, although some works have examined the potential of some miRNAs to differentiate among MM histotypes and to distinguish MM from lung carcinoma, data were insufficient for statistical analysis.

The traditional vote-counting approach highlighted huge expression discrepancies that failed to identify miRNA signatures that could distinguish specimens from MM or asbestos-exposed subjects from those of control individuals. The high variability among studies may be a contributing factor, since the MM samples included fresh/frozen biopsy tissue; formalin-fixed paraffin-embedded (FFPE) tissue; LCM tissue; macro-dissected tissue; tissue collected after treatment; plasma, serum, and blood cell fraction, and cell lines. Control samples also differed widely and included FFPE biopsies of healthy pleura, patient-matched non-neoplastic pleura, pericardium, lung, healthy lung tissue from asbestos-exposed subjects, tissue from a range of cancers, non-neoplastic proliferations, plasma/serum from healthy or exposed subjects, blood cell fraction of healthy/exposed subjects, immortalized cell lines, and normal human mesothelial cell cultures. Moreover, some studies comparing MM histotypes did not include a control group of normal samples. MiRNA quantification approaches also differed widely and included real-time quantitative PCR, qRT-PCR array, microarray, in situ hybridization-based assays, and variants thereof. Different platforms, statistics, qRT-PCR normalization methods, validation approaches, sample sizes, and other differences further hampered comparisons and reproducibility of miRNAs among studies. In addition, different criteria may have been applied in the different studies to evaluate exposed and non-exposed patients, leading to contrasting results. For instance, the widely different quality of data provided by functional evidence and patient reports of asbestos exposure clearly result in formation of groups that are not strictly comparable. Altogether, these variables are the cause of the numerous inconsistencies (Supplementary Table 2).

The suspicion that some of the deregulated miRNAs identified by the traditional vote count might be false positives prompted us to try to identify those having a key role in MM pathogenesis and asbestos-related malignancies.

#### Finding MM-associated miRNAs

To identify a miRNA signature for early diagnosis, the background noise was reduced by including only qRT-PCR-validated miRNAs obtained by comparison categories (a) MM tissue *vs*. normal or non-cancer tissue, and (c) MM blood samples *vs.* normal blood samples.

**Figure 2:**
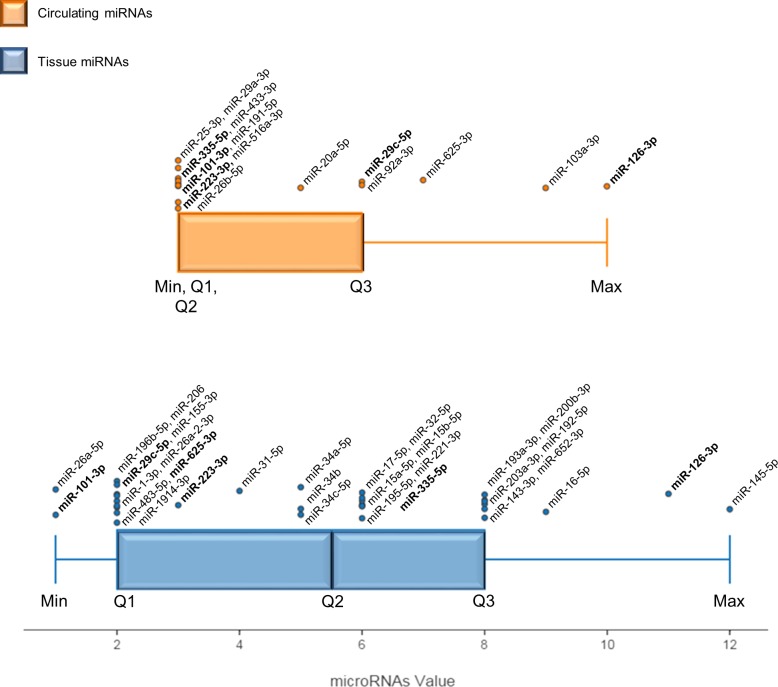
Box and whisker plot displaying MM-miRNA value distributions in tissue and the circulation The value of each miRNA is the sum of multiple features scored in the specially designed vote-counting method. Max and Q3 values identify the most significant miRNAs in blood and tissue. Each dot overlying the total distribution represents a miRNA and respective names are reported. MiRNAs in bold are described both in blood and tissue. The black bar represents the median of each distribution (Q2). Min: lowest value, Q1: lower quartile, Q2: median quartile, Q3: upper quartile, Max: highest value.

A qualitative meta-analysis was conducted to improve the results of the traditional vote-count, miRNA values were then calculated, and a box-whisker plot was used to represent quartile subdivision and miRNA distribution in tissues and blood samples (Figure 2). The miRNAs found in Max and Q3 were considered the most useful in distinguishing MM from healthy or asbestos-exposed subjects.

The method identified 9 miRNAs, *i.e.* miR-145-5p, miR-126-3p, miR-16-5p, miR-192-5p, miR-193a-3p, miR-200b-3p, miR-203-3p, miR-143-3p and miR-652-3p, as the most significant miRNAs in tissue (Figure 2). All 9 are down-regulated in MM compared with healthy subjects (Table 2); their downregulation in MM tissue was confirmed by more than one qRT-PCR series, as detailed in Supplementary Methods. Moreover, miR-145, miR-126, and miR-16-5p are reported in two different papers, and miR-192, miR-193a-3p, miR-200b, and miR-203 are included in the most numerous pool of MM samples (>100) analyzed to date (Table 2). A signature including these 9 miRNAs could thus have clinical relevance for MM.

**Table 2:** MM-miRNAs from qRT -PCR analyses employed in our qualitative meta-analysis

Accession number	miRBase ID	miRNAs	MM *vs*. non-cancer tissues					MM blood samples *vs*. blood healty samples					Gene family	Clustered miRNAs	Cytogenetic location
			No. of qRT-PCR	MM	H	D	Ref	No. of qRT-PCR	MM	H	D	Ref			
MIMAT0000416	**hsa-miR-1-3p**	miR-1	1	25	6	↓	[75]						mir-1	hsa-mir-1-2, hsa-mir-133a-1	20q13.33, 18q11.2
MIMAT0000099	**hsa-miR-101-3p**	miR-101	1	n.a.	n.a.	↓	[93]	1	14	10	↑	[91]	mir-101	hsa-mir-101-1, hsa-mir-3671	1p31.3, 9p24.1
MIMAT0000101	**hsa-miR-103a-3p**	miR-103 / miR-103a-3p						2	66	77	↓	[88], [89]	mir-103	hsa-mir-103a-2, hsa-mir-103b-2, hsa-mir-103b-1, hsa-mir-103a-1	20p13, 5q34
MIMAT0000445	**hsa-miR-126-3p**	miR-126	5	59	51	↓	[85], [77]	2	89	106	↓	[85], [86]	mir-126	/	9q34.3
MIMAT0000435	**hsa-miR-143-3p**	miR-143	4	32	24	↓	[77]						mir-143	hsa-mir-145, hsa-mir-143	5q32
MIMAT0000437	**hsa-miR-145-5p**	miR-145	6	74	74	↓	[76], [77]						mir-145	hsa-mir-143, hsa-mir-145	5q32
MIMAT0004658	**hsa-miR-155-3p**	miR-155*	1	25	6	↑	[75]						mir-155	/	21q21.3
MIMAT0000068	**hsa-miR-15a-5p**	miR-15a-5p	1	60	23	↓	[94]						mir-15	hsa-mir-15a, hsa-mir-16-1	13q14.2
MIMAT0000417	**hsa-miR-15b-5p**	miR-15b-5p	1	60	23	↓	[94]						mir-15	hsa-mir-15b, hsa-mir-16-2	3q25.33
MIMAT0000069	**hsa-miR-16-5p**	miR-16 / miR-16-5p	2	78	30	↓	[90], [94]						mir-15	hsa-mir-15a, hsa-mir-16-1, hsa-mir-15b, hsa-mir-16-2	13q14.2, 3q25.33
MIMAT0000070	**hsa-miR-17-5p**	miR-17-5p	1	32	24	↓	[83]						mir-17	hsa-mir-17, hsa-mir-18a, hsa-mir-19a, hsa-mir-20a, hsa-mir-19b-1, hsa-mir-92a-1	13q31.3
MIMAT0000440	**hsa-miR-191-5p**	miR-191						1	14	10	↓	[91]	mir-191	hsa-mir-191, hsa-mir-425	3p21.31
MIMAT0007890	**miR-1914-3p**	miR-1914-3p	1	18	7	↓	[90]						mir-1914	hsa-mir-647, hsa-mir-1914	20q13.33
MIMAT0000222	**hsa-miR-192-5p**	miR-192	2	120	23	↓	[99]						mir-192	hsa-mir-6750, hsa-mir-194-2, hsa-mir-192	11q13.1
MIMAT0000459	**hsa-miR-193a-3p**	miR-193a-3p	2	120	23	↓	[99]						mir-193	/	17q11.2
MIMAT0000461	**hsa-miR-195-5p**	miR-195-5p	1	60	23	↓	[94]						mir-15	hsa-mir-497, hsa-mir-195	17p13.1
MIMAT0001080	**hsa-miR-196b-5p**	miR-196b	1	18	7	↓	[90]						mir-196	/	7p15.2
MIMAT0000318	**hsa-miR-200b-3p**	miR-200b	2	120	23	↓	[99]						mir-8	hsa-mir-200a, hsa-mir-200b, hsa-mir-429	1p36.33
MIMAT0000264	**hsa-miR-203a-3p**	miR-203	2	120	23	↓	[99]						mir-203	hsa-mir-203a, hsa-mir-203b	14q32.33
MIMAT0000462	**hsa-miR-206**	miR-206	1	25	6	↓	[75]						mir-1	hsa-mir-206, hsa-mir-133b	6p12.2
MIMAT0000075	**hsa-miR-20a-5p**	miR-20a						1	23	25	↓	[88]	mir-17	hsa-mir-17, hsa-mir-18a, hsa-mir-19a, hsa-mir-20a, hsa-mir-19b-1, hsa-mir-92a-1	13q31.3
MIMAT0000278	**hsa-miR-221-3p**	miR-221	1	32	24	↑	[83]						mir-221	hsa-mir-222, hsa-mir-221	Xp11.3
MIMAT0000280	**hsa-miR-223-3p**	miR-223	2	17	6	↓	[98]	1	14	10	↓	[91]	mir-223	/	Xq12
MIMAT0000081	**hsa-miR-25-3p**	miR-25						1	14	10	↑	[91]	mir-25	hsa-mir-25, hsa-mir-93, hsa-mir-106b	7q22.1
MIMAT0000082	**hsa-miR-26a-5p**	miR-26a	1	n.a.	n.a.	↓	[93]						mir-26	/	3p22.2, 12q14.1
MIMAT0004681	**hsa-miR-26a-2-3p**	miR-26a-2-3p	1	18	7	↓	[90]						mir-26	/	12q14.1
MIMAT0000083	**hsa-miR-26b-5p**	miR-26b						1	14	10	↑	[91]	mir-26	/	2q35
MIMAT0000086	**hsa-miR-29a-3p**	miR-29a						1	14	10	↑	[91]	mir-29	hsa-mir-29a, hsa-mir-29b-1	7q32.3
MIMAT0004673	**hsa-miR-29c-5p**	miR-29c*	1	18	7	↓	[90]	1	45	24	↑	[90]	mir-29	hsa-mir-29b-2, hsa-mir-29c	1q32.2
MIMAT0000089	**hsa-miR-31-5p**	miR-31	1	25	20	↓	[97]						mir-31	/	9p21.3
MIMAT0000090	**hsa-miR-32-5p**	miR-32	1	27	27	↓	[85]						mir-32	/	9q31.3
MIMAT0000765	**hsa-miR-335-5p**	miR-335	1	27	27	↓	[85]	1	14	10	↑	[91]	mir-335	/	7q32.2
MIMAT0000255	**hsa-miR-34a-5p**	miR-34a	1	47	10	↓	[101]						mir-34	/	1p36.22, 1p36.22
MIMAT0004676/MIMAT0000685	**hsa-miR-34b-3p / hsa-miR-34b-5p**	miR-34b	1	47	10	↓	[101]						mir-34	hsa-mir-34b, hsa-mir-34c	11q23.1
MIMAT0000686	**hsa-miR-34c-5p**	miR-34c	1	47	10	↓	[101]						mir-34	hsa-mir-34b, hsa-mir-34c	11q23.1
MIMAT0001627	**hsa-miR-433-3p**	miR-433						1	14	10	↑	[91]	mir-433	hsa-mir-337, hsa-mir-665, hsa-mir-431, hsa-mir-433, hsa-mir-127, hsa-mir-432, hsa-mir-136	14q32.2
MIMAT0004761	**hsa-miR-483-5p**	miR-483-5p	1	25	6	↓	[75]						mir-483	/	11p15.5
MIMAT0006778	**hsa-miR-516a-3p (unclear)**	miR-516						1	14	10	↑	[91]	mir-515	hsa-mir-522, hsa-mir-519a-1, hsa-mir-527, hsa-mir-516a-1, hsa-mir-1283-2, hsa-mir-516a-2, hsa-mir-519a-2, hsa-mir-521-1, hsa-mir-519a-2	19q13.42
MIMAT0004808	**hsa-miR-625-3p**	miR-625-3p	1	18	7	↑	[90]	2	45	24	↑	[90]	mir-625	/	14q23.3
MIMAT0003322	**hsa-miR-652-3p**	miR-652	4	32	24	↓	[77]						mir-652	/	Xq23
MIMAT0000092	**hsa-miR-92a-3p**	miR-92a						1	45	24	↑	[90]	mir-25	hsa-mir-17, hsa-mir-18a, hsa-mir-19a, hsa-mir-20a, hsa-mir-19b-1, hsa-mir-92a-1, hsa-mir-106a, hsa-mir-18b, hsa-mir-20b, hsa-mir-19b-2, hsa-mir-92a-2, hsa-mir-363	13q31.3, Xq26.2

Note: Qualitative meta-analysis involved exclusively miRNAs analyzed by RT-PCR in tissue and blood samples. MiRNAs were ranked based on the total number of tumor and healthy samples involved and on the number of qRT-PCR assays performed. Accession number, miRNA unique identifier (ID), gene family, clustered miRNAs, and cytogenetic location are reported for each miRNA according to the last miRBase release (miRBase v21).

No. of qRT-PCR: total number of additional qRT-PCR assays described in the same paper and/or in different papers (*e.g.* qRT-PCR in screening set and qRT-PCR in validation set); MM: number of MM samples used in qRT-PCR analyses; H: number of non-cancer controls samples used in qRT-PCR analyses; D: miRNA deregulation trend in MM specimens compared with control samples (↑: up-regulated miRNAs and ↓: down-regulated miRNAs). Ref: references in parentheses are numbered according to the reference list.

Despite the small number of studies investigating circulating miRNAs, the large number of samples analyzed and of qRT-PCRs performed involved that several circulating miRNAs were found in Max and Q3 (Figure 2). In Max, miR-126-3p was described in two consecutive studies of MPM plasma and serum [85, 86], whereas miR-103a-3p was identified in the blood cellular fraction from MPM patients [88, 89]. Moreover, miR-625-3p upregulation was confirmed in multiple-step analysis of plasma and serum with low-level hemolysis [90].

The most significant tissue and circulating miRNAs identified here could have clinical relevance and could be specifically involved in the pathogenetic process triggered by asbestos exposure.

#### Assessment of the diagnostic potential of tissue and circulating MM miRNAs

Since miRNAs are abnormally expressed in several cancers and pathophysiological conditions, it is useful to establish whether promising miRNAs are MM-specific or are also found in other conditions. The 41 qRT-PCR-validated miRNAs, consisting of 26 miRNAs validated in tissues, 9 in blood and 6 miRNAs in both kind of samples (Table 2), were thus compared with those most frequently reported as biomarkers in other cancers and in subjects exposed to environmental pollution.

**Figure 3:**
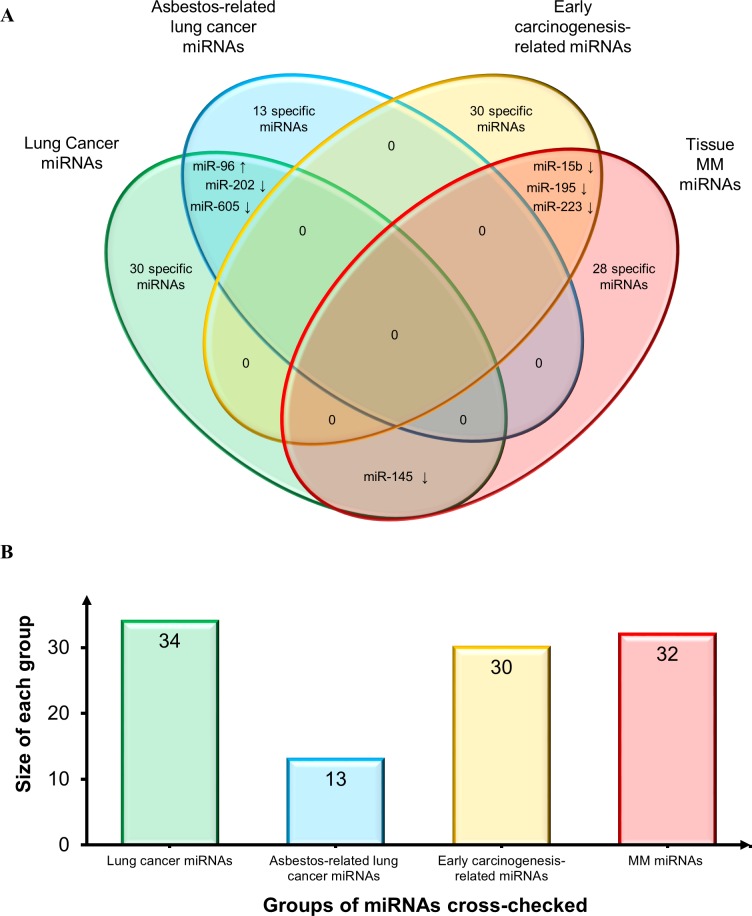
Comparison of MM miRNAs and those reported in asbestos-exposed subjects and asbestos-related lung cancers **A.** Venn diagram comparing tissue MM miRNAs and three pools of miRNAs identified by Nymark *et al.* [70]: 13 novel “lung cancer miRNAs”, “asbestos-related lung cancer miRNAs”, and “early carcinogenesis-related miRNAs”. Shared miRNAs are reported at intersections. MiRNAs were identified by comparing not only miRNA identifiers, but also deregulation trends, depicted by arrows (↑: up-regulated miRNAs and ↓ down-regulated miRNAs). **B.** Histogram displays groups of miRNAs cross-checked and size of each group.

First, we compared the MM tissue miRNAs to those reported by Nymark *et al*. in the sole miRNA profiling study retrieved by our search, which compared lung cancer and corresponding normal tissue from subjects with a history of high asbestos exposure *vs.* non-exposed patients and control (non-neoplastic) lung tissue specimens [70]. The authors found 13 novel miRNAs and divided them into 3 sets: “lung cancer miRNAs”, “asbestos-related lung cancer miRNAs”, and “early carcinogenesis-related miRNAs”. Their comparison with our MM tissue miRNAs (Venn diagram in Figure 3) highlighted some common miRNAs. However, based on the direction of deregulation, there were only 3 miRNAs shared by “lung cancer miRNAs” and “asbestos-related lung cancer” (miR-202 and miR-605, down-regulated, and miR-96, up-regulated); 3 down-regulated miRNAs shared by “MM miRNAs” and “early carcinogenesis-related miRNAs” (miR-15b, miR-195 and miR-223); and down-regulated miR-145 in “MM miRNAs” and “lung cancer-related miRNAs”.

**Figure 4:**
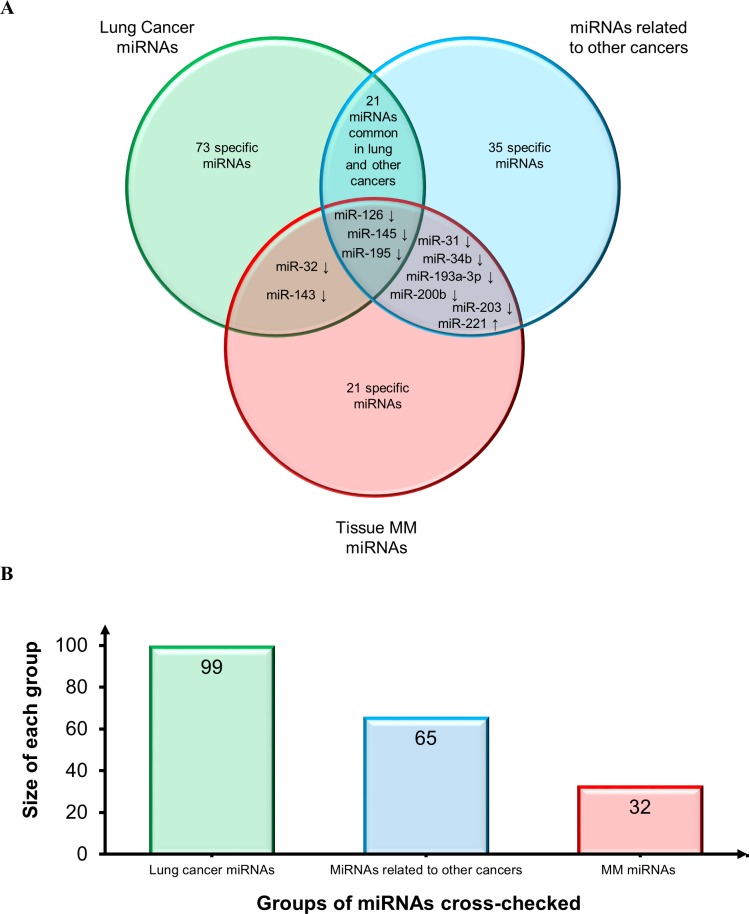
Assessment of diagnostic potential of tissue MM miRNAs **A.** Venn diagram comparing tissue MM miRNAs and the miRNAs most frequently reported in lung cancer tissues and in a range of tissues from other cancers. Shared miRNAs are reported at intersections. MiRNAs were identified by comparing not only miRNA identifiers, but also deregulation trends, depicted by arrows (↑: up-regulated miRNAs and ↓ down-regulated miRNAs). **B.** Histogram displays groups of miRNAs cross-checked and size of each group.

The same MM miRNAs were then compared with those most frequently described in lung cancer tissue [126–128] and in tissues from other cancers including glioblastoma, head and neck, breast, liver, gastric, pancreatic, cervical, ovarian, prostatic, colorectal (CRC), and bladder cancer [128]. The Venn diagram reported in Figure 4 shows consistent downregulation of miR-126, miR-145, and miR-195, thus confirming their involvement in the cancer phenotype also based on their detection at other sites besides lung: miR-126, gastric and prostate cancer; miR-145, breast and bladder cancer; and miR-195, bladder cancer. Moreover, miR-143 and miR-32 are down-regulated both in lung cancer and MM. MiRNAs shared by MM and other cancers include the following down-regulated miRNAs: miR-31 (gastric, prostate, bladder, head and neck cancer, CRC); miR-34b (breast cancer), miR-193a-3p (CRC), miR-200b (ovarian cancer), and miR-203 (pancreatic, cervical and prostate cancer). MiR-221 (gastric, cervical, and prostate cancer and glioblastoma) is the sole shared up-regulated miRNA. Since several other candidate biomarkers were shared by lung and other cancers and showed the same trend, they are all likely to play a role in the molecular pathways that are disrupted in cancer. Moreover, given the different sites of the malignancies, the cross-check suggested a biomarker role for most of them (Figure 4).

**Figure 5:**
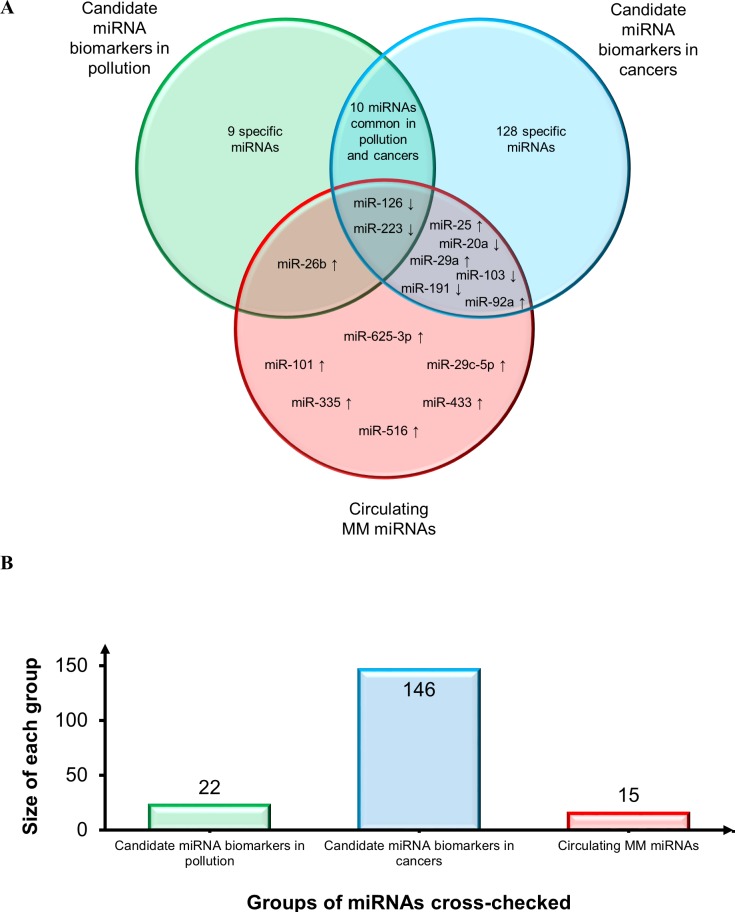
Assessment of diagnostic potential and specificity of circulating MM-miRNAs **A.** Venn diagram showing circulating MM miRNAs, the circulating miRNAs most frequently reported in a variety of conditions and in a pool of different cancers particularly enriched in lung cancer miRNAs, and deregulated miRNAs responsive to personal or environmental pollution exposure including smoking, chemicals and air pollution. Common miRNA names are reported at the intersections. MiRNAs were identified by comparing not only miRNA identifiers, but also deregulation trends, depicted by arrows (↑: up-regulated miRNAs and ↓ down-regulated miRNAs). **B.** Histogram displays groups of miRNAs cross-checked and size of each group.

**Figure 6:**
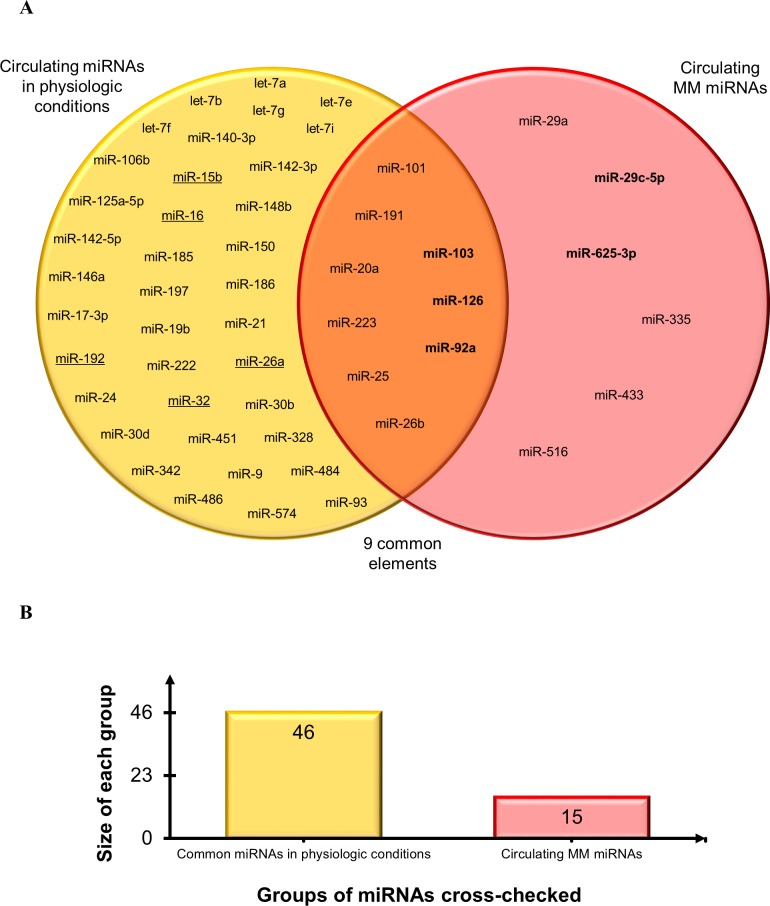
Comparison of circulating MM-miRNAs and the miRNAs most frequently reported in healthy individuals **A.** Venn diagram comparing the datasets. Common miRNAs are reported at intersections. MiRNAs in bold are the most significant circulating MM miRNAs. Underlined miRNAs are also listed among the deregulated miRNAs analyzed in MM tissues. **B.** Histogram displays groups of miRNAs cross-checked and size of each group.

Secondly, to establish the diagnostic potential and specificity of the circulating miRNAs, these were checked against i) the circulating miRNA biomarkers most frequently reported in a variety of conditions, including a pool of different cancers that are particularly enriched in lung cancer miRNAs [127–129], and ii) the deregulated miRNAs associated with personal or environmental exposure to noxious stimuli including cigarette smoking, chemicals, and polluted air [64]. The results of this comparison, reported in Figure 5, demonstrate that relatively few miRNAs can be considered as common biomarkers of MM, pollution, and cancer. Based on the direction of deregulation, only miR-126 and miR-223 are shared by all sets. MiR-126 has been described as a diagnostic marker in non-small cell lung carcinoma (NSCLC) [127] and, interestingly, it is down-regulated in leukocytes exposed to particulate matter, black carbon, organic carbon, and sulfate ions (SO_4_^2-^) [64]. Low miR-223 levels are found in subjects exposed to tobacco smoke and in those with acute myeloid leukemia (AML) [64], sepsis [129], prostate cancer, and leukemia [128]. MiR-103 and miR-191 are down-regulated common miRNAs in MM and in serum from NSCLC patients *vs.* healthy smokers, whereas miR-20a is down-regulated in plasma from lung cancer surgery patients *vs.* healthy controls [127]. MiR-25 is up-regulated in breast, liver, bladder [128], and lung cancer [129]; miR-29a in CRC, ovarian [128,129], breast cancer [128], and NSCLC [127], and miR-92a in CRC, ovarian, prostate, and liver cancer [128,129]. MiR-26b is up-regulated in MM and in a miRNA pool related to pollution exposure, and shows expression changes in cord blood due to arsenic exposure [64]. Several miRNAs were seen to be shared by pollution exposure and cancer patients, but considering that many cancers are related to pollution the finding is not unexpected. Finally, to test whether our pool of circulating MM miRNAs are tumor-specific or commonly detected in the circulation, the MM miRNAs were checked against the circulating miRNAs described most frequently in healthy individuals [129]. The diagram in Figure 6 documents that 9/15 circulating MM miRNAs are commonly found in healthy individuals, but are deregulated in MM, whereas 6/15 include up-regulated miR-625-3p and 5 other miRNAs that are found exclusively in the MM set.

#### Functional and statistical evaluation of the most significant circulating MM miRNAs

To identify the most significant circulating MM miRNAs with the strongest diagnostic potential and function, we queried the miRandola database, a comprehensive, manually curated classification of extracellular circulating miRNAs [130, 131], where cell-free miRNAs are divided into four carrier-based categories: miRNA-Ago2, miRNA-exosome, miRNA-HDL, and when the specific carrier is unknown, miRNA-circulating [130, 131]. Moreover, the miRNAexpress tool in miRandola provides a systematic comparison of the expression profiles of cellular and extracellular miRNAs [130, 131] and allows identification of the miRNAs that are specifically expressed in tissue/cells, those unique to the circulation, and those shared by cells and the circulation. The MiRandola and miRNAexpress outputs for miR-103a-3p, miR-126-3p, miR-29c-5p, miR-92a-3p, and miR-625-3p are reported in Table 3, where the sample types investigated, the carrier identified, any validated targets, the biomarker value in other diseases, and miRNAexpress data are reported for each miRNA.

**Table 3:** miRandola and miRNAExpress analysis of the most significant circulating miRNAs

Circulating MM miRs	miR-126-3p ↓	miR-103a-3p ↓	miR-625-3p ↑	miR-29c-5p ↑	miR-92a-3p ↑
**Samples**	Plasma/serum	T cells/Dendritic cells	Plasma/serum	Serum/T cells	Plasma/serum/T cells/Dendritic cells
**Specific carrier identified**	Exosome	Ago2/Exosome	HDL (normal)/Exosomes (prostate cancer)	Exosomes	Ago2/Exosome
**Validated target and function**	**VCAM1** (vascular cell adhesion molecule 1)	**ICOS** (inducible T-cell co-stimulator), **SERBP1** (SERPINE1 MRNA Binding Protein 1), **FBXW11** (F-box and WD repeat domain containing 11)	Unknown	Unknown	Unknown
**Potential biomarker in other diseases**	**Yes:** metastatic colorectal cancer^*^, prostate cancer^*^, urothelial bladder cancer^*^, osteoarthritis, type 2 diabetes, acute myocardial infarction, endurance exercise, stable/unstable angina	Unknown	**Yes:** low levels in NSCLC	Unknown	**Yes:** high level in gastric cancer, colorectal carcinomas, and hepatitis C infection
**miRNAExpress output**	Cells and circulation	Cells and circulation	Circulation	Cells	Cells and circulation

**Note:** information retrieved in miRandola is organized into four groups: miRNA-Ago2, miRNA-HDL, miRNA-exosomes and miRNA-circulating. The latter is used here when Ago2, exosome and HDL are not described in the paper.

* = up-regulated in these cancers.

**Figure 7:**
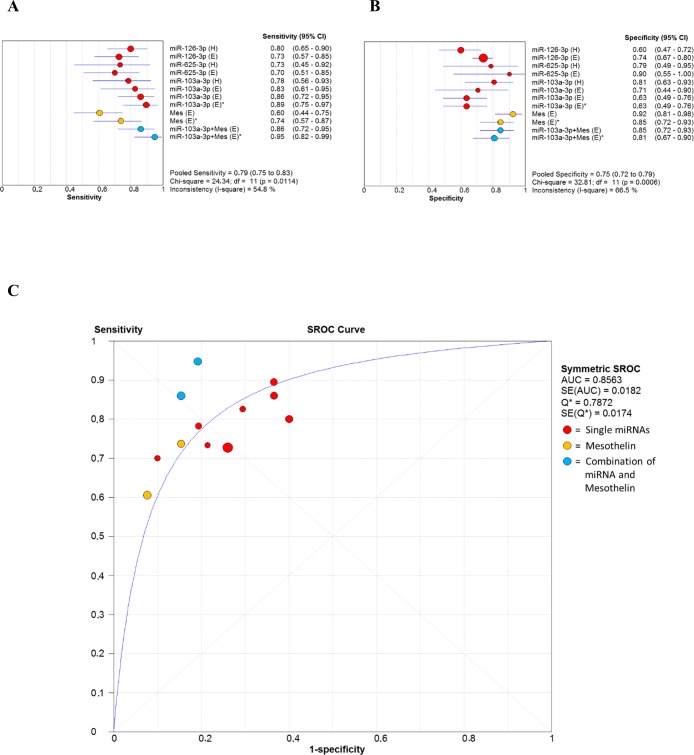
Forest plot of sensitivities (6A) and specificities (6B) of miRNAs, mesothelin and their combination in the diagnosis of MM The point estimate is bounded by a 95% confidence interval (CI). Forest plots do not contain a pooled summary due to the high heterogeneity of data. The plots are useful to obtain a general overview of the accuracy estimates from each miRNAs, mesothelin or their combination. (**6C**) Summary receiver operating characteristics (SROC) curve for the diagnosis of MM through circulating miRNA, mesothelin and a combination of both biomarkers. AUC area under curve, Q* index, SE standard error.

Since accuracy estimates were reported for miR-103a-3p, miR-126-3p, and miR-625-3p from the original studies, forest plots for sensitivities (Figure 7A) and specificities (Figure 7B) were drawn to obtain a general overview of the accuracy estimates for the three miRNAs, mesothelin and their combination. As we found significant heterogeneity between studies, the random effects model was applied. The SROC curve for the biomarkers investigated was shown in Figure 7C. The pooled area under curve (AUC) of SROC was 0.8563, suggesting promising accuracy for circulating miRNA in diagnosing MM.

Table 4 also shows the pooled results for diagnostic accuracy in different subgroups. Similar results are observed in the diagnosis of MM using different reference groups (healthy subjects or asbestos exposed subjects). However, subgroup analysis based on the combination of miRNA and mesothelin (*i.e.* miR-103a-3p and mesothelin in Table 4) suggested that a multiple-biomarkers assay showed superior diagnostic properties than assay based on mesothelin alone or single miRNAs for MM detection in both general population and asbestos exposed subjects.

**Table 4:** Subgroup analyses for the diagnosis of MM reporting summary estimates of diagnostic criteria and their 95% confidence intervals

Analysis	Sensitivity(95% CI)	Specificity(95% CI)	Positive LR(95% CI)	Negative LR(95% CI)	DOR(95% CI)
**Single-miRNA**					
Reference group: healthy subjects	0.78[0.68-0.87]	0.69 [0.59-0.77]	2.68 [1.61-4.47]	0.32 [0.21-0.48]	8.28[4.19-16.36]
Reference group: asbestos exposed subjects	0.79 [0.71-0.85]	0.72 [0.67-0.78]	2.82 [2.22-3.58]	0.3 [0.21-0.42]	10.25[6.05-17.38]
Combination of miRNA and mesothelin^*^					
Reference group: asbestos exposed subjects	0.90 [0.82-0.96]	0.88[0.74-0.89]	5.23 [3.42-8.02]	0.12 [0.06-0.23]	46.65[18.94-114.9]

CI confidence interval, LR likelihood ratio, DOR diagnostic odds ratio.

* Combination of miR-103a-3p and mesothelin as proposed by Weber *et al*. [89].

## DISCUSSION

A mounting number of studies have been documenting the involvement of miRNAs in carcinogenesis and molecular changes driven by pollution exposure, suggesting the scope for using them as diagnostic markers as well as therapeutic targets in a variety of diseases. Finding biomarkers capable of predicting MM development in subjects with occupational and/or environmental asbestos exposure would have huge implications, especially considering that MM diagnosis is invasive and that there is still no effective cure. However, despite intense research efforts under way in several laboratories, finding consistency is hampered by differences in miRNA profiling methods and in the technological approaches adopted. As a result, the identification of minimally invasive, inexpensive diagnostic/prognostic tests for MM is still elusive. Given that, profiling studies provide a myriad miRNAs, many of which may have no clinical relevance, a meta-analysis of miRNA datasets would yield important findings [75].

To our knowledge a meta-analysis of miRNAs related to asbestos exposure and MM has never been performed, despite the value of secondary data analysis in highlighting high-quality evidence and in providing guidance when experimental studies disagree. Data extrapolation is a crucial phase of systematic research, and including comparable numerical values is a precondition for a meta-analysis. The paucity of online-available raw datasets on MM miRNA expression made it impossible to apply a broad statistical approach to the data provided by the literature search. Only a few of the 39 papers retrieved by our search were based on extensive profiling and even fewer provided raw datasets. To address this key limitation, we felt that the most rational and transparent approach would be apply a vote-counting strategy. Yet, after the traditional vote-counting approach yielded a highly heterogeneous pool of 214 miRNAs (due to the broad diversity of asbestos-related malignancies, analytical methods, and study designs), it became apparent that handling such a vast dataset would require a more refined approach. However, its overview provided two important pieces of information: i) that most MM miRNAs were down-regulated compared with the respective control groups (Supplementary Table 1); and ii) that miRNA expression in blood samples and biopsies showed a certain consistency, whereas expression in cell lines largely differed from tissue data (Table 1 and Supplementary Table 1). This suggests that the use of cell line data should be confined to functional assays. Numerous miRNAs were found to be deregulated in more than one study. Even though some did not show a clear trend (Table 1), they are nonetheless likely to be involved in the disruption of key ARD and MM pathways. It will be the task of future functional studies to clarify their role and influence.

To pare down the 213-strong pool and exclude spurious miRNAs, only high-confidence miRNAs validated by qRT-PCR were retained and subjected to further analysis. This reduced the candidate pool to a dataset of 41 MM-related miRNAs (Table 2) that could then be used as starting points to discover upstream and downstream molecules involved in MM pathogenesis. Next, to enable more accurate data assessment, a qualitative meta-analysis of the miRNA pool was conducted by applying an *ad hoc* devised vote-counting strategy. This approach is based on a scoring method that took into account the direction of deregulation and four selected features: i) the number of qRT-PCR assays conducted to assess each miRNA; ii) the total number of MM samples, iii) the total number of normal samples used; and iv) the number of studies reporting each miRNA as deregulated. The circulating and tissue miRNAs thus identified therefore had a highly probable biomarker value and deserve further investigation as “mesomiRs” (*mesothelioma-associated miRNAs*).

### Tissue MM miRNA signature

Examination of the 41-miRNA dataset clearly demonstrated a general trend toward downregulation of tissue miRNAs (Table 2), confirming the hypothesis that the miRNAs showing significant underexpression in cancer tissue may have a tumor suppressing function, whereas those that are up-regulated may be tumor promoters (oncomiRs) [132, 133]. The hypothesis is also supported by reports of the identification of several tumor-suppressing miRNAs and oncomiRs through their modulation of gene expression [134–136]. Moreover, since MM is characterized by chromosome instability (*i.e.* 1p36, 9p21, 3p, 4q, 6q, 14q32, 17p13, and 22q12 deletions), and instability has been related to underexpression of tumor suppressor genes [100, 137, 138], the miRNAs targeting them are also conceivably candidate biomarkers for MM. In contrast, chromosome gains have been reported at 1q, 5p15, 7p12, 8q24 and 17q [137, 138]. Several significant miRNAs identified in our study map to the above loci or to fragile genomic regions (Table 2), and might be down- or up-regulated due to accumulation of acquired chromosomal losses or gains and other copy number changes.

The downregulation of miR-145-5p, miR-143-3p, miR-126-3p, miR-652-3p, and miR-16-5p, and the upregulation of miR-625-3p, highlighted by the qualitative meta-analysis, agree with the chromosomal instability and epigenetic modifications described in MM. MiR-145 showed the highest score (Figure 2), and its downregulation in MM and other lung cancers could be explained by its role in carcinogenesis. The miR-145 and the miR-143 family are clustered miRNAs involved in p53 downstream regulation [77]. Both map to the 5q32 locus, which is prone to hypermethylation in mesothelioma and mesothelioma cell lines [76]. The hypermethylation of this locus probably accounts for their low expression found in MM samples.

MiR-126-3p, miR-625-3p, and miR-16-5p map respectively to loci 9q34.3, 14q23.3, and 13q14.2, which are prone to deletion and copy number changes [139–141]. Downregulation of the miR-15 family has been described in other solid tumors like lung, colon, ovary, and prostate, and the gene locus has been shown to be deleted in more than half of B cell chronic lymphocytic leukemias (CLLs) [60, 77]; in the latter, the miR-15a/16-1 cluster targets the oncogene BCL2 and functions as a tumor suppressor [142]. MiR-126-3p tends to be affected in any disease causing micro- or macro-vascular damage, inflammation, and aging [52, 53, 143]. The most recent findings suggest a role for it in regulating the amino acid transporter LAT1 in MM cells [77]. A key role for it in controlling oxidative stress in MM has also been proposed, and would be in line with the suppression of miR-126-3p seen in MM patients [110]. Further work is needed to elucidate the biological roles of miR-625-3p and miR-652-3p in carcinogenesis.

Besides chromosome instability, miRNA downregulation could also be explained by two key features of MM tumors: the hypoxic phenotype and the high levels of epidermal growth factor receptor (EGFR) [144]. In the cytoplasm, precursor miRNAs are cleaved by the ribonuclease Dicer to their mature length and are then loaded on Ago2 proteins; formation of the RISC complex leads to achievement of their functional form. In hypoxic conditions EGFR is internalized into intracellular vesicles, where it phosphorylates cytoplasmic Ago2, reducing precursor binding to Dicer, hence the number of mature miRNAs [144].

Moreover, analysis of the miRNAs found in Q2 disclosed interesting links with those in Max and Q3. Downregulation of miR-15a-5p, miR-15b-5p, and miR-195-5p has been reported by a study of more than 50 MM samples; significantly, all these miRNAs belong to the same gene family or to clustered miRNAs, such as miR-16-5p. Since only one study has described miRNA behavior in asbestos-related lung cancers [70], it is impossible to draw any conclusions, but the finding that miR-15b and miR-195 are related to early carcinogenesis in asbestos-exposed subjects (Figure 3) adds to the relevance of the miR-15 family. Interestingly, the phase I MesomiR I trial has found considerable metabolic and radiological response in a MPM patient using a novel targeted miRNA-based treatment based on delivery vehicles packaged with miR-16-based mimics (denominated TargomiRs) [145]. Though preliminary, these preclinical data are promising and confirm that miRNA mimics and anti-miRs may be able to restore gene networks.

Methylation-induced silencing has been shown by various functional studies to induce downregulation of clustered miR-34b and miR-34c [103–106, 146]. Several other validated miRNAs belong to clusters or to the same family, *i.e.* most of mir-1, mir-17, mir-25, mir-26, mir-29 family members, and share similar expression trends. These findings confirm the involvement of all these miRNAs in the cancer phenotype and their key role in derailed pathways.

Assessment of the diagnostic potential of the most significant tissue miRNAs (Figure 4) disclosed that the “MM”, “lung cancer” and “other cancers” sets share only 3 miRNAs. Few other candidate MM biomarkers are shared by “lung cancer” or “other cancers”, but the strong disparity at the sites of onset of the relevant malignancies makes them discriminating for MM.

The qualitative meta-analysis, conducted with a specially devised vote-counting method, identified miRNAs with close relevance to asbestos-related carcinogenesis. It has recently been suggested that a four-miRNA classifier (miR-126-3p, miR-143-3p, miR-145-5p, miR-652-3p) can be applied to differentiate MM from non-neoplastic tissue samples with sensitivity and specificity [77]. It is reasonable to hypothesize that its performance could be improved by adding the five tissue miRNAs identified by our qualitative meta-analysis - miR-16-5p, miR-192-5p, miR-193a-3p, miR-200b-3p, and miR-203a-3p - thus giving rise to an MM-miR signature. Analysis of all nine miRNAs might provide more accurate diagnostic information, and conceivably even divide patients based on relative miRNA expression and rate of cancer progression. The hypothesis that multiple miRNAs might be more accurate is supported by the polyclonal nature of MM and other ARDs, since the carcinogenic effect of mineral fibers involves that multiple cells undergo malignant transformation, and each clone may develop and expand its own distinctive set of molecular alterations [147].

### Circulating miRNAs as biomarkers in MM and asbestos-exposed patients

Circulating miRNAs are ideal biomarkers since they are non-invasive, stable, they vary little in the general population, and are not expensive to analyze.

The five circulating miRNAs found by our qualitative meta-analysis include four detected in plasma/serum - miR-126-3p, miR-29c-5p, miR-92a-3p and miR-625-3p - and one - miR-103a-3p - detected in the cellular fraction of peripheral blood (Figure 2). Numerous circulating miRNAs showing clinically significant properties have also been detected in patients with several different conditions and in individuals exposed to environmental pollutants. To test the diagnostic potential and specificity of miR-126-3p, miR-29c-5p, miR-92a-3p, miR-625-3p, and miR-103a-3p, we compared three circulating miRNA sets -“MM-related miRNAs”, “cancer-related miRNAs”, and “miRNAs related to pollution exposure” (Figure 5) - and found that miR-126 downregulation is shared by all three sets. Even though its downregulation has been reported in NSCLC compared with healthy smokers/healthy controls [127], miR-126 is significantly more down-regulated in MM than in NSCLC patients/healthy controls, and can also stratify MM patients by length of survival [86].

In the “pollution exposure” set, miR-126 downregulation has been detected in leukocytes as an effect of particulate matter, black carbon, organic carbon, and SO_4_^2-^ [64]; the latter ions are found in the atmosphere as aerosols produced by fossil fuel and biomass combustion. A similar trend of downregulation is induced by mineral fibers and particulate matter containing asbestos.

Down-regulated miR-103 is shared by “MM-related miRNAs” and “cancer-related miRNAs”, but the different type of specimens analyzed (respectively cellular fraction of peripheral blood and serum) makes it unique to MM (Figure 5).

Although experimental data suggest that miRNAs released into body fluids do not necessarily reflect their abundance in the cell of origin [130], we also tested whether our pool of circulating MM miRNAs are tumor-specific or else they are miRNAs commonly detected in the circulation. We thus compared the fifteen circulating MM miRNAs with those commonly found in healthy individuals. The Venn diagram in Figure 6 shows that nine commonly detected miRNAs are instead down-regulated in MM, and include miR-103 and miR-126 (the latter showing the same trend also in tissue). In contrast, six miRNAs, including up-regulated miR-625-3p and miR-29c-5p, are specific of the MM set. MiR-29c-5p has prognostic value, since higher expression is associated with a favorable prognosis in MM patients [74], reinforcing its value as a candidate MM biomarker.

Interestingly, some miRNAs that are deregulated in tissue from MM as well as from asbestos-exposed individuals, *i.e.* miR-15b, miR-16, miR-192, miR-26a, and miR-32, belong to the group of physiological circulating miRNAs, but they have never been analyzed in plasma/serum from MM patients. In contrast, low miR-16 has been detected in the cellular fraction of peripheral blood from asbestos-exposed individuals (Supplementary Table 1) [88].

Since convincing evidence has highlighted that Ago proteins, HDL, and exosomes transport and deliver miRNAs to recipient cells having different regulatory requirements, we also investigated the specific carriers of circulating MM miRNAs, hypothesizing that vesicle-, Ago2-, and HDL-associated miRNAs may originate from cells reflecting cell type-specific expression and release mechanisms. A MiRandola interrogation demonstrated that in plasma and serum miR-126-3p is mainly associated with exosomes (Table 3). Tumor-derived exosomes function as shuttles in the cross-talk between tumor microenvironment and distant cell targets. OncomiRs are actively secreted by cancer cells and promote tumor formation and progression by acting on extracellular matrix remodeling, inducing angiogenesis, and regulating stromal cells and stem cell niches [66, 148]. MiR-126-3p has tumor-suppressing functions [149], and its loss promotes tumor cell formation, migration, and invasion, and prevents anti-tumor immune response. Vascular cell adhesion molecule 1 (VCAM1) is a validated target of miR-126-3p (Table 3). VCAM1 is important in cell-cell recognition, it appears to function in leukocyte-endothelial cell adhesion and signal transduction, and may play a pathophysiological role both in the immune response and in leukocyte migration to the sites of inflammation. Response reprogramming involves active transfer of exosomal miRNAs between immune cells [150]. Notably, the findings that miR-126-3p tends to be up-regulated in other cancers (Table 3) and that exosome-derived miRNAs share the miRNA profile of their tissue of origin [151] confirm its potential biomarker value in MM. Vascular endothelial growth factor (VEGF) is another specific target of miR-126-3p [152]. Its upregulation plays a critical role in tumor progression [153] and inversely proportional levels of VEGF-miR-126-3p are found in blood from MPM patients [85]. Given its tumor-suppressing functions, miR-126-3p is also a potential therapeutic target in MM. Moreover, re-expression of miR-126 reduced tumor cell migration and invasion in colon cancer [154], and both mature forms of mir-126 hindered metastasis progression by reducing inflammatory monocyte and mesenchymal stem cell recruitment to the site of the primary tumors [155].

After a study of ovarian cancer specimens [87] suggested that all neoplasms may generate a unique miRNA fingerprint in the peripheral blood cell fraction, a similar investigation of samples from MM patients proposed a biomarker role for miR-103a-3p [88, 89]. Since our search retrieved no further studies using this approach, the available data are insufficient to judge whether miRNA fingerprints in the peripheral blood cell fraction reflect a cancer-specific or a blood cell-based phenomenon. The miRNA profile of the peripheral blood cell fraction is likely largely conditioned by endogenous miRNAs of peripheral blood mononuclear cells (PBMCs). Moreover T, B, and dendritic immune cells have been shown to have a different exosomal miRNA cargo compared with their parent cells, due to exosomal cross-talk between regulatory RNAs and recipient cells during immune synapsis [156]. In this context, low levels of specific miRNAs or the genetic alteration of key components in miRNA processing can compromise the immune response and lead to tumor formation [156]. Examination of the characteristics of miR-103a-3p in the miRandola database showed that miR-103a-3p is associated with Ago2 proteins and exosomes in T cells and dendritic cell samples (Table 3). Inhalation of asbestos fibers has been found to impair immune response and tumor immunity by affecting immunocompetent cells [157]. In particular, asbestos exposure suppresses human naïve CD8+ lymphocyte differentiation into cytotoxic T lymphocytes (CTLs), which recognize and kill non-self target cells [158]. Analysis of the number and characteristics of PBMCs has demonstrated that their total number is lower in MM and asbestos-exposed patients than in healthy individuals, and that CD8+ lymphocytes suffer from functional impairment both in MM and in asbestos-exposed patients [159]. Whereas MM is associated with diminished tumor immunity, exposed patients retain an effective immune function [159]. These findings are in line with the downregulation of miR-103a-3p described in MM patients. Our miRandola analysis identified some validated targets that reinforce these findings (Table 3): i) inducible T-cell co-stimulator (ICOS), which plays an important role in cell-cell signaling, immune response, and regulation of cell proliferation; ii) SERPINE1 (SERBP1), an mRNA binding protein that may play a role in regulating mRNA stability; iii) FBXW11 (F-box and WD repeat domain containing 11), which is involved in the ubiquitination and subsequent proteasomal degradation of target proteins, participates in Wnt signaling, and may function in the intra-S-phase checkpoint in oxidative stress. MiRandola does not consider miR-103a-3p as a potential biomarker in other diseases; it would be interesting to know whether it has a similar behavior in PBMCs and the whole cell fraction of peripheral blood, because according to previous evidence miR-10b expression in PBMCs can discriminate NSCLC patients from healthy subjects with high sensitivity and specificity [160]; iv) miR-625-3p is carried by HDL and exosomes, and has biomarker value in serum from NSCL patients when it is significantly down-regulated compared with healthy individuals [161]; v) high miR-625-3p levels have been reported in plasma/serum from MM patients, and miRNAexpress analysis has identified it as circulation-specific, whereas miR-126-3p and miR-103a-3p are also commonly found in cells; this has also been confirmed by a study addressing the impact of cellular miRNAs on circulating miRNA biomarkers [162]. Although the function and targets of miR-625-3p are still unclear, our data identify it as a potentially MM-specific miRNA; vi), miR-29c-5p is up-regulated both in cells and the circulation; according to the miRandola database it is predominantly associated with cells; this agrees with the finding that it has prognostic potential in MM tissue [74], since its level can segregate patients by histotype, and higher expression correlates with a more favorable prognosis; however its expression in plasma/serum is less significant [90]; finally vii) miR-92a-3p is up-regulated in cells and the circulation; high levels have been reported in gastric cancer, CRC, and hepatitis C infection, where it has been found to have biomarker potential. It is therefore non-specific for MM and asbestos exposure and is less significant as a potential MM biomarker (Table 3).

MiR-126-3p, miR-103a-3p, miR-29c-5p, miR-92a-3p, and miR-625-3p are likely released by cancer cells *via* different pathways to serve different functions. The hypothesis is supported by the report that they showed marked differences in some serum fractions from the same individual and between healthy individuals and cancer patients [163]. This suggests that miRNA stratification in ultracentrifuged samples may reflect their different shuttles, and stresses the need for assessing miRNA levels in the context of their carriers when trying to discover diagnostic biomarkers of cancer.

### An MM multimarker signature and future prospects

MiR-126-3p, miR-103a-3p, and miR-625-3p have provided some interesting results in distinguishing MM from healthy or asbestos-exposed patients, whereas the diagnostic ability of miR-29c-5p and miR-92a-3p requires further evaluation. MiR-126-3p and miR-103a-3p have demonstrated a sensitivity of 73-80% and 83-89%, respectively, but their relatively low specificity (60-74% and 63-71%, respectively) prevents their clinical application as standalone biomarkers [85, 88, 89]. In contrast, miR-625-3p has shown a specificity of 78-90% and a sensitivity of 70-73% (Figure 7A-7B) [90]. Several proteins have been proposed as MM biomarkers over the past few years. Mesothelin is currently the most widely used, thanks to its high specificity (*ca.* 89%) and despite its low sensitivity (58%) [164]. According to recent evidence, HMGB1 serum levels and the relative levels of its different isoforms (hyper-acetylated and non-acetylated HMGB1) can distinguish MM patients from asbestos-exposed individuals and unexposed controls with 100% sensitivity and specificity, outperforming existing biomarkers (mesothelin, fibulin-3, and osteopontin), whereas HMGB1 combined with fibulin-3 improves differential diagnosis [13]. Notably, harnessing markers from different molecular classes has been shown to provide high diagnostic performances in MM.

To our knowledge, a parallel analysis of miRNAs and HMGB1 has never been performed, but combined analysis of mesothelin and either miR-126-3p or miR-103a-3p has improved the specificity and sensitivity of each marker alone in distinguishing asbestos-exposed from MM patients (Figure 7A-7B-7C) [85, 89]. This finding, and the report that a combination of circulating miRNAs rather than a single miRNA biomarker increased early diagnostic performance [165], suggest that an MM-multimarker signature including miR-126-3p, miR-103a-3p, miR-625-3p, and mesothelin would maximize the effectiveness of asbestos-exposed patient monitoring for early detection of the switch to carcinogenesis. Pooled results for diagnostic accuracy of the combination miR-103a-3p and mesothelin revealed the highest value of sensitivity 90% (0.82-0.96), specificity 82.7% (0.74-0.89) and DOR 46.65 (18.94-114.9) (Table 4).

Moreover, it would be useful to assess the feasibility of using different fractions of whole blood in monitoring high-risk patients. In fact, the plasma/serum levels of circulating miRNAs may be masked by other miRNAs released by hematopoietic cells [162], and differences in blood cell counts, sample hemolysis, and cargo discarded after cell death are all causes of variations in miRNA levels [166, 167]. The problem could be addressed by a standard protocol. Simultaneous analysis of miR-103a-3p in the cellular fraction and of miR-126-3p, miR-625-3p, and mesothelin in plasma/serum from the same sample in conjunction with blood cell counts and assessment of hemolysis might provide such a protocol [166, 167]. Assessment of its results would provide solid evidence about the value of this approach in early diagnosis, and possibly stratification based on pathophysiological condition and cancer risk.

## CONCLUSIONS

**Figure 8:**
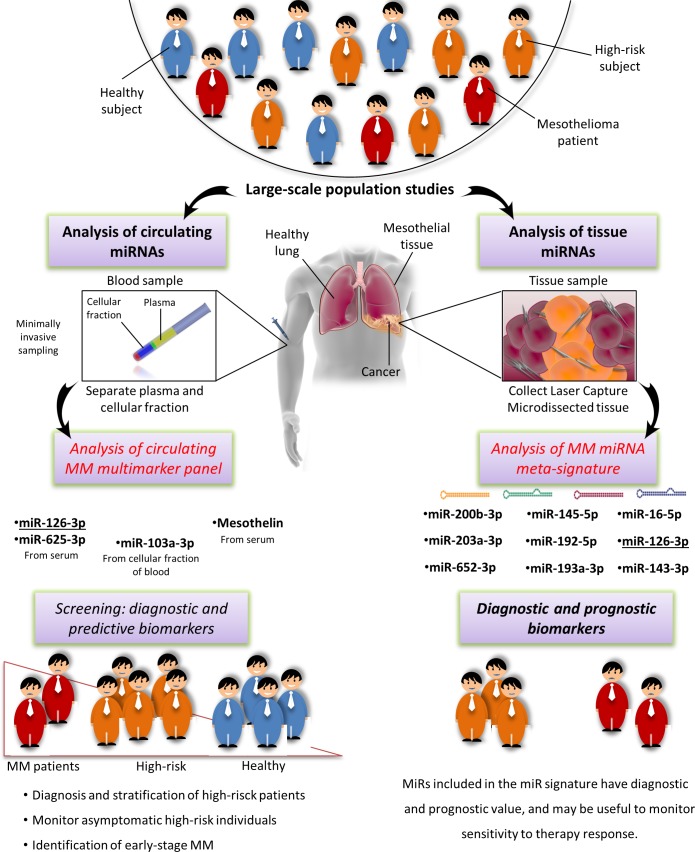
Schematic drawing illustrating summary findings On left side: potential pivotal role of miRNAs in the minimally invasive surveillance of high risk patients and early diagnosis of MM cases. On right side: diagnostic and prognostic potential of the most important tissue miRNAs. Parts of the images were adapted from (http://www.somersault1824.com/).

It is believed that the morbidity and mortality caused by asbestos exposure will peak in the next decade [168]. Minimally invasive monitoring approaches are thus urgently needed, both to extend patient lifespan and to preserve their quality of life. All published data confirm the key importance of miRNAs in MM diagnosis, prognosis and treatment, highlighting the need for more specific circulating biomarkers of asbestos exposure. The method illustrated above is a useful approach to identify consistent miRNAs that can be used as MM biomarkers when raw data are not available. A recent assessment of the value of vote-counting methods has disclosed that the number of supporting studies combined with the size of the sample tested by RT-qPCR provides sound biomarker ranking [169]. The process adopted in our qualitative meta-analysis has yielded a reliable list of mesomiRs candidates. Large-scale, standardized validation is required to establish whether the tissue miRNA meta-signature (miR-16-5p, miR-126-3p, miR-143-3p, miR-145-5p, miR-192-5p, miR-193a-3p, miR-200b-3p, miR-203a-3p, and miR-652-3p) and the MM multimarker panel (miR-126-3p, miR-103a-3p, miR-625-3p, and mesothelin) proposed herein are capable of delivering accurate diagnoses and identifying high-risk patients (Figure 8). Furthermore, formation of exposed and non-exposed groups requires accurate assessment of exposure. Computed tomography and chest x-rays remain the more sensitive approaches to evaluate objective clinical parameters of exposure [11, 12, 170]. In the absence of detectable physiological changes, exposure intensity should be determined using *ad hoc* questionnaires [171, 172]. Besides their potential as clinically relevant biomarkers, the mesomiRs identified are a panel of consistently deregulated and highly significant molecules that should thoroughly be investigated to assess their involvement in the onset and progression of disease triggered by mineral fiber exposure. Here, we provide a framework and rationale for similar future investigations. A greater understanding of the cellular origin of circulating miRNAs will pave the way for the use of this exciting new class of analytes as cancer biomarkers.

## MATERIALS AND METHODS

### Literature search and screening

The PubMed, GEODataSet [173, 174] and EMBL-EBI ArrayExpress [175] databases were searched using the terms “microRNA”, “mesothelioma”, “asbestos”, “asbestosis” and all conceivable combinations of their synonyms (last accessed on 29 September 2015). Pre-established inclusion and exclusion criteria, listed in Table 5, were applied to screen query outputs.

**Table 5:** Search methodology and inclusion / exclusion criteria

Key words and Mesh terms used in PubMed, GEO DataSet and EBI ArrayExpress queries	Inclusion Criteria	Exclusion Criteria
**microRNA, microRNAs, miRNA, miRNAs, miR, miRs, malignant mesothelioma, asbestos, asbestos exposure**	Papers reporting miRNA profiling in MM and all types of asbestos exposure; papers reporting deregulation of single or multiple miRNAs in subjects with MM and asbestos exposure.	Papers not in English, duplicates, reviews. Paper describing only functional assays were considered in the sytematic review but excluded from the qualitative meta-analysis.

**Figure 9:**
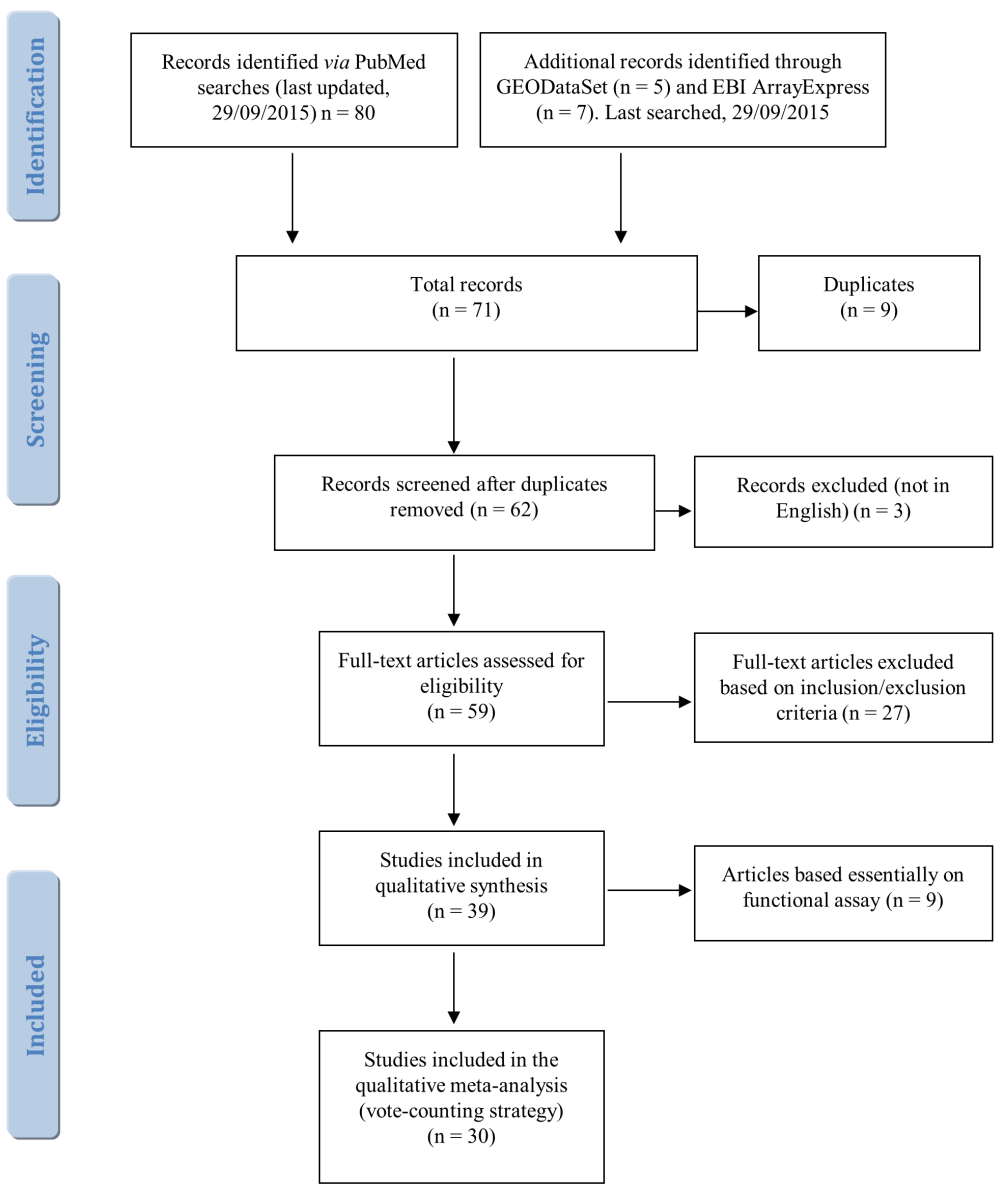
PRISMA Flow Diagram showing the selection process for the systematic review and qualitative meta-analysis

As shown in the PRISMA flow diagram (Figure 9), the search retrieved 80 papers and 39 datasets. Application of inclusion/exclusion criteria left 39 articles reporting miRNA deregulation in MM. Nine of these papers were based essentially on functional assays and were reviewed for the sake of completeness; the other 30 studies were subjected to a qualitative meta-analysis. The assessment of potential biases in the review process is reported in Supplementary Methods, Supplementary Table 3 and Supplementary Table 4.

### Data extraction and ranking

The full text, supplementary material, author, year of publication, study design, number of specimens investigated, tumor content and histology, methodological approach, GEO accession number, and relevant findings were extracted from each paper and are listed in Supplementary Table 2. The studies described 4 categories of comparisons: (a) MM tissue *vs*. normal or non-cancer tissue; (b) MM tissue *vs.* other cancer tissues; (c) MM blood samples *vs.* normal blood samples; and (d) MM cell lines *vs.* normal cell lines. The 213 miRNAs reported to be deregulated in specimens from MM and asbestos-exposed subjects compared with control samples were divided based on their trend of deregulation, and are reported in Supplementary Table 1. The miRNAs that had been validated by quantitative real-time PCR (qRT-PCR) were extracted, whereas all the other miRNAs were discarded. To narrow down the sample further, data that had been obtained by comparing miRNAs found in MM and other cancer types (b), data from cell lines (d); data obtained from comparison of different MM histotypes; and data from studies that did not report clear trend information for the miRNAs were excluded. This left only the 41 qRT-PCR-validated miRNAs that had been obtained by comparing (a) MM tissue *vs*. normal or non-cancer tissue and (c) MM blood samples *vs.* normal blood samples. These miRNAs are reported in Table 2 with their unique identifier (ID) and accession number, as available. Names were standardized according to the latest miRBase release (miRBase v21, June 2014 available at http://www.mirbase.org/) [176–180]. Any ambiguity regarding miRNA identity was solved using miRBase Tracker [181]. For each miRNA, gene family, clustered miRNAs, and cytogenetic locations are also reported.

### Vote-counting methods

Data were analyzed by a two-step approach. In the first step, a traditional vote-counting method was applied to the dataset of all deregulated miRNAs (Supplementary Table 1) to extract the miRNAs that had been reported most frequently in the largest number of studies and comparison categories (a, b, c, d; see under 5.2. Data extraction and ranking).

In the second step, a more stringent, specially devised vote-counting strategy was applied to the 41 miRNAs that had been validated by qRT-PCR (Table 2). Detailed information about this strategy is reported in Supplementary Methods. Results are reported as a box-whisker plot, where each dot represents a miRNA (Figure 2). The miRNAs belonging to groups Q3 and Max were considered as the most significant. Quartile ranking and box-whisker plot were obtained using Microsoft Excel and Plotly (https://plot.ly/).

### Statistical analysis

Statistical analysis of the most significant circulating miRNAs was undertaken utilizing Meta-DiSc 1.4 software [182]. The bivariate meta-analysis model was employed to summarize the sensitivity, specificity, and generate the bivariate summary receiver operator characteristic (SROC) curve with their corresponding 95% CIs among the studies using circulating miRNAs as biomarker for MM diagnosis. Due to high heterogeneity of the dataset, data pooling of sensitivity, specificity, diagnostic odds ratio (DOR), positive likelihood ratio (PLR), and negative likelihood ratio (NLR) was performed in omogeneous subgroups defined a priori [182, 183]. Cochran's Q test and inconsistency index (I^2^) test were employed to trace potential sources of study heterogeneity. *P* < 0.01 for Cochran's Q test, or I^2^ > 50%, all indicated an existence of significant heterogeneity [184]. When significant heterogeneity existed for sensitivity and specificity, the random effect model was employed.

### Bioinformatic analysis and functional investigation

The 41 miRNAs that had been validated by qRT-PCR (Table 2) were cross-checked with the miRNAs previously described as biomarkers and/or potential biomarkers in multiple pathophysiological conditions and in individuals exposed to environmental pollution using jvenn, an interactive Venn diagram viewer [185]. Since most reports use the earlier nomenclature, the earlier miRNA identifiers were used in this comparison, whereas the newer identifiers were used where multiple names might raise confusion.

As a further confirmation of their role and diagnostic potential, the tissue and circulating miRNAs identified by the meta-analysis were then checked against the miRandola database, a comprehensive manually curated classification of different extracellular circulating non-coding RNAs [130, 131]. Sequence alignment of SV40-encoded miRNas *vs.* human miRNAs was performed using the BLASTN search algorithm in miRBase (http://www.mirbase.org/).

## SUPPLEMENTARY MATERIAL










