# Supplementary material for: Diagnostic value of microRNAs in asbestos exposure and malignant mesothelioma: systematic review and qualitative meta-analysis
Source: Oncotarget. 2016 Jun 1;7(36):58606–37. doi: 10.18632/oncotarget.9686 (PMC5295457; doi:10.18632/oncotarget.9686)
Supplement: Supplementary file 1 [file oncotarget-07-58606-s001.pdf]

# Diagnostic value of microRNAs in asbestos exposure and malignant mesothelioma: systematic review and qualitative meta-analysis

**Supplementary Material**

**Methods**

## 1. Potential biases in the review process

The study was conducted in accordance with the PRISMA checklist [1], a system that improves the clarity and transparency of the systematic review approach. The PRISMA checklist has been developed for randomized trials [2], but it can also be useful as a basis for reporting systematic reviews of other kinds of research [1]. The 11-item AMSTAR (assessment of multiple systematic reviews) tool was also applied, to evaluate the methodological quality of systematic reviews [3]. The PRISMA and AMSTAR checklists are shown in **Supplementary Table 1** and **Supplementary Table 2**, respectively. Even with all these precautions, the possibility of publication bias remains; for example manuscripts that are suitable for publication in scientific journals are more likely to report statistically significant data [4–6]. Due to the small size of studies investigating diagnostic value of circulating miRNAs in MM, we did not evaluated publication bias using a funnel plot.

## 2. Details about the specially devised vote-counting strategy

Our scoring method was established *a priori* by three investigators. The method considers the direction of deregulation as well as the following 4 features: the number of studies reporting each miRNA as deregulated; the total number of MM samples; the total number of normal samples used; and the number of qRT-PCR series conducted to assess each miRNA. The latter characteristic has been taken into account because some authors have carried out the qRT-PCR analysis in different cohorts of samples (*e.g.* Discovery set, Training set and Validation set [79], see study design in

**Table 2**) and therefore performing more than one qRT-PCR series to evaluate miRNAs expression in the same study.

A value was attributed to each miRNA by assigning a score for each of the 4 features. We adopted quartiles, which divide an ordered data series into four equal parts, to set up a five-point scale for the number of samples used. In brief, each feature was given a score based on the quartile of the distribution it occupied: 0, the lowest value (Min); 1, the lower quartile (Q1); 2, the median quartile (Q2); 3, the upper quartile (Q3); and 4, the highest value (Max). The quartile rankings of each feature were calculated for (a) and (c) comparison categories, *i.e.* MM tissue *vs*. normal or non-cancer tissue and (c) MM blood samples *vs.* normal blood samples. However, since quartiles are typically calculated for lists with a minimum of 20 items, the approach could be used only for the number of MM or normal samples used. Thus, a 0-4 scale was used to assign a score to the number of qRT-PCR assays performed: 1 qRT-PCR, 0; 2 qRT-PCRs, 1; 4 qRT-PCRs, 2; 5 qRT-PCRs, 3; and 6 qRT-PCRs, 4. Finally, for the number of studies reporting the same miRNA, 1 point was awarded for report in one paper and 2 points for report in two or more papers. The 4 scores were added to obtain a final score for each miRNA, and quartiles were again calculated from their distribution. The following **Tables** (**A** and **B**) display the scoring scheme for tissue miRNAs and circulating miRNAs, respectively. Results are reported as a box-whisker plot, where each dot represents a miRNA (**Figure 2**). The miRNAs belonging to groups Q3 and Max were considered as the most significant.

**Table A.** Scoring table of tissue MM-miRNAs from qRT-PCR analyses.

| Accession number | miRBase ID | miRNAs | No. of qRT-PCR | qRT-PCR score | MM | MM score | H | H score | Ref | Ref score | miRNA Vote |
|---|---|---|---|---|---|---|---|---|---|---|---|
| MIMAT0000416 | **hsa-miR-1-3p** | miR-1 | 1 | 0 | 25 | 1 | 6 | 0 | [75] | 1 | **2** |
| MIMAT0000099 | **hsa-miR-101-3p** | miR-101 | 1 | 0 | n.a. | 0 | n.a. | 0 | [93] | 1 | **1** |
| MIMAT0000445 | **hsa-miR-126-3p** | miR-126 | 5 | 3 | 59 | 3 | 51 | 3 | [85], [77] | 2 | **11** |
| MIMAT0000435 | **hsa-miR-143-3p** | miR-143 | 4 | 2 | 32 | 2 | 24 | 3 | [77] | 1 | **8** |
| MIMAT0000437 | **hsa-miR-145-5p** | miR-145 | 6 | 4 | 74 | 3 | 74 | 3 | [76], [77] | 2 | **12** |
| MIMAT0004658 | **hsa-miR-155-3p** | miR-155* | 1 | 0 | 25 | 1 | 6 | 0 | [75] | 1 | **2** |
| MIMAT0000068 | **hsa-miR-15a-5p** | miR-15a-5p | 1 | 0 | 60 | 3 | 23 | 2 | [94] | 1 | **6** |
| MIMAT0000417 | **hsa-miR-15b-5p** | miR-15b-5p | 1 | 0 | 60 | 3 | 23 | 2 | [94] | 1 | **6** |
| MIMAT0000069 | **hsa-miR-16-5p** | miR-16 / miR-16-5p | 2 | 1 | 78 | 3 | 30 | 3 | [90], [94] | 2 | **9** |
| MIMAT0000070 | **hsa-miR-17-5p** | miR-17-5p | 1 | 0 | 32 | 2 | 24 | 3 | [83] | 1 | **6** |
| MIMAT0007890 | **miR-1914-3p** | miR-1914-3p | 1 | 0 | 18 | 1 | 7 | 0 | [90] | 1 | **2** |
| MIMAT0000222 | **hsa-miR-192-5p** | miR-192 | 2 | 1 | 120 | 4 | 23 | 2 | [99] | 1 | **8** |
| MIMAT0000459 | **hsa-miR-193a-3p** | miR-193a-3p | 2 | 1 | 120 | 4 | 23 | 2 | [99] | 1 | **8** |
| MIMAT0000461 | **hsa-miR-195-5p** | miR-195-5p | 1 | 0 | 60 | 3 | 23 | 2 | [94] | 1 | **6** |
| MIMAT0001080 | **hsa-miR-196b-5p** | miR-196b | 1 | 0 | 18 | 1 | 7 | 0 | [90] | 1 | **2** |
| MIMAT0000318 | **hsa-miR-200b-3p** | miR-200b | 2 | 1 | 120 | 4 | 23 | 2 | [99] | 1 | **8** |
| MIMAT0000264 | **hsa-miR-203a-3p** | miR-203 | 2 | 1 | 120 | 4 | 23 | 2 | [99] | 1 | **8** |
| MIMAT0000462 | **hsa-miR-206** | miR-206 | 1 | 0 | 25 | 1 | 6 | 0 | [75] | 1 | **2** |
| MIMAT0000278 | **hsa-miR-221-3p** | miR-221 | 1 | 0 | 32 | 2 | 24 | 3 | [83] | 1 | **6** |
| MIMAT0000280 | **hsa-miR-223-3p** | miR-223 | 2 | 1 | 17 | 1 | 6 | 0 | [98] | 1 | **3** |
| MIMAT0000082 | **hsa-miR-26a-5p** | miR-26a | 1 | 0 | n.a. | 0 | n.a. | 0 | [93] | 1 | **1** |
| MIMAT0004681 | **hsa-miR-26a-2-3p** | miR-26a-2-3p | 1 | 0 | 18 | 1 | 7 | 0 | [90] | 1 | **2** |
| MIMAT0004673 | **hsa-miR-29c-5p** | miR-29c* | 1 | 0 | 18 | 1 | 7 | 0 | [90] | 1 | **2** |
| MIMAT0000089 | **hsa-miR-31-5p** | miR-31 | 1 | 0 | 25 | 1 | 20 | 2 | [97] | 1 | **4** |
| MIMAT0000090 | **hsa-miR-32-5p** | miR-32 | 1 | 0 | 27 | 2 | 27 | 3 | [85] | 1 | **6** |
| MIMAT0000765 | **hsa-miR-335-5p** | miR-335 | 1 | 0 | 27 | 2 | 27 | 3 | [85] | 1 | **6** |
| MIMAT0000255 | **hsa-miR-34a-5p** | miR-34a | 1 | 0 | 47 | 2 | 10 | 2 | [101] | 1 | **5** |
| MIMAT0004676/ MIMAT0000685 | **hsa-miR-34b-3p / hsa-miR-34b-5p** | miR-34b | 1 | 0 | 47 | 2 | 10 | 2 | [101] | 1 | **5** |
| MIMAT0000686 | **hsa-miR-34c-5p** | miR-34c | 1 | 0 | 47 | 2 | 10 | 2 | [101] | 1 | **5** |
| MIMAT0004761 | **hsa-miR-483-5p** | miR-483-5p | 1 | 0 | 25 | 1 | 6 | 0 | [75] | 1 | **2** |
| MIMAT0004808 | **hsa-miR-625-3p** | miR-625-3p | 1 | 0 | 18 | 1 | 7 | 0 | [90] | 1 | **2** |
| MIMAT0003322 | **hsa-miR-652-3p** | miR-652 | 4 | 2 | 32 | 2 | 24 | 3 | [77] | 1 | **8** |

**Note:** MiRNAs were ranked based on the total number of tumor and healthy samples involved and on the number of qRT-PCR assays performed. Accession number and miRNA unique identifier (ID) are reported for each miRNA according to the last miRBase release (miRBase v21).

**No. of qRT-PCR:** total number of additional qRT-PCR assays described in the same paper and/or in different papers and correspondig score (**qRT-PCR score**); **MM:** number of MM samples used

in qRT-PCR analyses and correspondig score (**MM score**); **H:** number of non-cancer controls samples used in qRT-PCR analyses and correspondig score (**H score**); **Ref**: number of references for each miRNAs and corresponding score (**Ref. score**), references in parentheses are numbered according to the reference list; **miRNA Vote:** final score for each miRNA obtained by adding up the scores of the 4 features.

**Table B.** Scoring table of circulating MM-miRNAs from qRT-PCR analyses.

| Accession number | miRBase ID | miRNAs | No. of qRT-PCR | qRT-PCR score | MM | MM score | H | H score | Ref | Ref score | miRNA Vote |
|---|---|---|---|---|---|---|---|---|---|---|---|
| MIMAT0000099 | hsa-miR-101-3p | miR-101 | 1 | 0 | 14 | 0 | 10 | 2 | [91] | 1 | **3** |
| MIMAT0000101 | hsa-miR-103a-3p | miR-103 / miR-103a-3p | 2 | 1 | 66 | 3 | 77 | 3 | [88], [89] | 2 | **9** |
| MIMAT0000445 | hsa-miR-126-3p | miR-126 | 2 | 1 | 89 | 3 | 106 | 4 | [85], [86] | 2 | **10** |
| MIMAT0000440 | hsa-miR-191-5p | miR-191 | 1 | 0 | 14 | 0 | 10 | 2 | [91] | 1 | **3** |
| MIMAT0000075 | hsa-miR-20a-5p | miR-20a | 1 | 0 | 23 | 1 | 25 | 3 | [88] | 1 | **5** |
| MIMAT0000280 | hsa-miR-223-3p | miR-223 | 1 | 0 | 14 | 0 | 10 | 2 | [91] | 1 | **3** |
| MIMAT0000081 | hsa-miR-25-3p | miR-25 | 1 | 0 | 14 | 0 | 10 | 2 | [91] | 1 | **3** |
| MIMAT0000083 | hsa-miR-26b-5p | miR-26b | 1 | 0 | 14 | 0 | 10 | 2 | [91] | 1 | **3** |
| MIMAT0000086 | hsa-miR-29a-3p | miR-29a | 1 | 0 | 14 | 0 | 10 | 2 | [91] | 1 | **3** |
| MIMAT0004673 | hsa-miR-29c-5p | miR-29c* | 1 | 0 | 45 | 2 | 24 | 3 | [90] | 1 | **6** |
| MIMAT0000765 | hsa-miR-335-5p | miR-335 | 1 | 0 | 14 | 0 | 10 | 2 | [91] | 1 | **3** |
| MIMAT0001627 | hsa-miR-433-3p | miR-433 | 1 | 0 | 14 | 0 | 10 | 2 | [91] | 1 | **3** |
| MIMAT0006778 | hsa-miR-516a-3p (unclear) | miR-516 | 1 | 0 | 14 | 0 | 10 | 2 | [91] | 1 | **3** |
| MIMAT0004808 | hsa-miR-625-3p | miR-625-3p | 2 | 1 | 45 | 2 | 24 | 3 | [90] | 1 | **7** |
| MIMAT0000092 | hsa-miR-92a-3p | miR-92a | 1 | 0 | 45 | 2 | 24 | 3 | [90] | 1 | **6** |

**Note:** MiRNAs were ranked based on the total number of tumor and healthy samples involved and on the number of qRT-PCR assays performed. Accession number and miRNA unique identifier (ID) are reported for each miRNA according to the last miRBase release (miRBase v21).

**No. of qRT-PCR:** total number of additional qRT-PCR assays described in the same paper and/or in different papers and correspondig score (**qRT-PCR score**); **MM:** number of MM samples used in qRT-PCR analyses and correspondig score (**MM score**); **H:** number of non-cancer controls

samples used in qRT-PCR analyses and correspondig score (**H score**); **Ref:** number of references for each miRNAs and corresponding score (**Ref. score**), references in parentheses are numbered according to the reference list; **miRNA Vote:** final score for each miRNA obtained by adding up the scores of the 4 features.

# REFERENCES


1. Moher D, Liberati A, Tetzlaff J, Altman DG. Preferred reporting items for systematic reviews and meta-analyses: the PRISMA statement. J Clin Epidemiol. 2009; 62(10):1006–12.

2. Liberati A, Altman DG, Tetzlaff J, Mulrow C, Gøtzsche PC, Ioannidis JPA, Clarke M, Devereaux PJ, Kleijnen J, Moher D. The PRISMA statement for reporting systematic reviews and meta-analyses of studies that evaluate health care interventions: explanation and elaboration. PLoS Med. 2009; 6(7):e1000100.

3. Shea BJ, Grimshaw JM, Wells GA, Boers M, Andersson N, Hamel C, Porter AC, Tugwell P, Moher D, Bouter LM. Development of AMSTAR: a measurement tool to assess the methodological quality of systematic reviews. BMC Med Res Methodol. 2007; 7:10.

4. Dickersin K, Min YI. NIH clinical trials and publication bias. Online J Curr Clin Trials. 1993; Doc No 50:[4967 words; 53 paragraphs].

5. Dwan K, Gamble C, Williamson PR, Kirkham JJ. Systematic review of the empirical evidence of study publication bias and outcome reporting bias - an updated review. PLoS One. 2013; 8(7):e66844.

6. Stern JM, Simes RJ. Publication bias: evidence of delayed publication in a cohort study of clinical research projects. BMJ. 1997; 315(7109):640–5.
