# Supplementary material for: Diagnostic value of microRNAs in asbestos exposure and malignant mesothelioma: systematic review and qualitative meta-analysis
Source: Oncotarget. 2016 Jun 1;7(36):58606–37. doi: 10.18632/oncotarget.9686 (PMC5295457; doi:10.18632/oncotarget.9686)
Supplement: Supplementary file 3 [file oncotarget-07-58606-s003.pdf]

Supplementary Table 2. Detailed overview of the studies included in the analysis.

Author, year of publication and reference	Characteristics of tumor samples	Tumor content	Characteristics of control samples	Study phase	qRT-PCR Platform	Microarray platform	Relevant findings	GEO Database and MIAME accession numbers
**Guled M *et al.* 2009 [71]**	Fresh tissue: MM (Total, 17: 10 epith, 1 sarc, 5 biph, 1 other)	30-90%	Fresh tissue: pericardium (Total, 1)	Screening	/	Agilent's miRNA microarray system (V.2) - Agilent Technologies	12 miRNAs exclusively expressed in MM samples, 9 miRNAs exclusively expressed in control samples. (Analysis of association with histopathological subtypes)	/
**Busacca S *et al.*2010 [82]**	Cell lines: MPP-89 (spindle-shaped), REN (epithelial-like)	/	Cell lines: HMC-TERT (immortalized human mesothelial cells-telomerase reverse transcriptase) (Total, 1)	Screening and validation	TaqMan MicroRNA assays - Applied Biosystem (Ref Gene: U6, calibrator sample)	miRCURY locked nucleic acid [LNA] microarray kit, V.8 - Exiqon	65 dysregulated miRNAs and 10 miRNAs validated by qRT-PCR	A-MEXP-1260, E-MEXP-1699
	FFPE tissue: MM (Total, 24: 8 epith, 8 sarc, 8 biph)	>80%	/	/	TaqMan MicroRNA assays - Applied Biosystem (Ref Gene: U6, calibrator sample)	/	10 miRNAs (analysis of association with histopathological subtypes)	
**Pass HI *et al.* 2010 [74]**	Fresh-frozen tissue: MPM (Total, 142: 81 epith, 48 other)	129/142 good RNA quality	/	Training set, test set	/	Nexterion® Slide	Analysis of association with histopathological subtypes and prognosis.	/
	16 MPM samples (8 good prognosis, 8 poor prognosis)	/	/	Validation	qRT-PCR (Ref Gene: miR-181a, miR-let-7c, miR-193a-5p, miR-27b, miR-339-5p)	/	Elevated miR-29c*is associated with longer survival	
	Cell lines: HP1, HP3, H2373, H2452, H2591, H2595, H2596, H2461	/	Cell lines: NYU-590.2 (primary mesothelial cell culture), LP9 (TERT-immortalized mesothelial cell line), SV40-transformed mesothelial cell line Met-5A	/	qRT-PCR (Ref Gene: n.a.)	n.a.	MiR-29c* is downregulated in MM cell lines	
**Ivanov SV *et al.* 2010 [84]**	Cell lines: HP1, HP3, H2373, H2452, H2591, H2595, H2596, H2461	/	Cell lines: NYU-590.2 (primary mesothelial cell culture), LP9 (TERT-immortalized mesothelial cell line), SV40-transformed mesothelial cell line Met-5A	Analysis of chromosome deletion	Loop RT-PCR technology (Ref Gene: n.a.)	Nexterion® Slide	Loss of miR-31 has a pro-tumorigenic effect in MM	/
**Benjamin H *et al.* 2010 [73]**	FFPE tissue: MPM (Total, 47: 29 epith, 6 sarc, 6 biph, 6 other)	Used 33/47	FFPE tissues: carcinomas (Total, 210/259)	Biomarker discovery, training phases	qRT-PCR (Ref Gene: U6)	Nexterion® Slide	Search for miRNAs capable of differentiating MM from carcinoma	/
	FFPE tissue: MPM (Used n=14/47)	≥ 50%	FFPE tissues: carcinomas (Total, 49/259)	Validation phase	qRT-PCR (Ref Gene: U6)	/	MiR-193-3p, miR-200c, and miR-192 are useful to differentiate MPM from other	

							pleural malignancies (sensitivity 100%, specificity 94%)	
**Gee GV *et al.* 2010 [72]**	Fresh tissue: MPM (Total, 15 other)	/	Fresh tissues: lung adenocarcinoma (Total, 10)	Screening	/	Affimetrix gene chip miRNA microarrays (TGmirV1b520432f)	Mesothelial-specific downregulation of 7 miRNAs in MPM compared with lung adenocarcinoma	/
	Fresh tissue: MPM (Total, 100: 39 epith, 10 sarc, 19 biph, 32 other)	/	Fresh tissue: lung adenocarcinoma (Total, 32) and normal lung tissue (Total, 4)	Validation	TaqMan MicroRNA assays - Applied Biosystem (Ref Gene: RNU44, RNU48)	/	MPM can be distinguished from lung adenocarcinoma by downregulation of 7 miRs	
**Balatti V *et al.* 2011 [81]**	Cell lines: MSTO-211H, MPP-89, IST-MES2,NCI-H2052, NCI-H28 (Total, 5)	/	Cell lines: HMCs (primary mesothelial cell culture: 4N, 6N, 13N, 16N, 26N) (Total, 5)	Screening	/	Agilent Human microRNA microarray G4470A - Agilent Technologies	MiRNAs found to be dysregulated may be potential MPM targets	/
				Validation	TaqMan MicroRNA assays - Applied Biosystem (Ref Gene: U6)	/		
**Santarelli L *et al.* 2011 [85]**	Fresh tissue: MPM (Total, 10: 9 epith, 1 sarc)	/	Fresh tissue: adjacent non-cancerous tissues (Total, 5)	Screening	/	miRNA qRT-PCR array MAH-102A - SABiosciences (Ref Gene:	Several downregulated miRs in MPM specimens	/
	Fresh tissue: MPM (Total, 27: 23 epith, 1 sarc, 3 biph)	/	FFPE tissue: adjacent non-cancerous tissues (Total, 27)	Larger-scale analysis	TaqMan MicroRNA assays - Applied Biosystem (Ref Gene: U6)	/	MiR-126 allows to discriminate cancer from non-malignant tissue	
	Serum: MPM patients (Total, 44: 30 epith, 6 sarc, 8 biph) and healthy asbestos-exposed subjects (Total, 196)	/	Serum: healthy subjects (Total, 50)	Validation	TaqMan MicroRNA assays - Applied Biosystem (Ref Gene: U6)	/	MiR-126, in association with SMRPs, was proposed as a marker for early detection of MPM	
**Kubo T *et al.* 2011 [101]**	Cell lines: NCI-H28, NCI-H290, NCI-H2052, NCI-H2452, HP1, MSTO-211H (Total, 6)	/	Cell cultures: non-malignant mesothelial primary cultures (Total, 2)	Analysis of expression status and methylation	qRT-PCR (Ref Gene: Expression levels of 2 non-malignant mesothelial cells mixed)	/	MiR-34b/c expression was reduced in all methylated MPM cell lines and tumors	/
	Fresh tissue: MPM (Total, 47: 32 epith, 4 sarc, 10 biph, 1 lymph) only 10 samples used for miRNA expression analysis	/	Fresh frozen tissue: non-neoplastic pleura from lung cancer patients (Total, 10)		qRT-PCR (Ref Gene: Expression levels of two non-malignant mesothelial cells mixed)	/		
**Fassina A *et al.* 2011 [92]**	FFPE tissue: 74 MPM and 35 peritoneal MM. Total, 109: 58 epith (35 pleural, 23 peritoneal), 25 sarc (18 pl+7 p), 26 biph (21 pl+5 p)	/	/	Analysis of miR-205	*In Situ* Hybridization and NCodeTM miRNA qRT-PCR - Invitrogen (Ref Gene: RNU6B)	/	MiR-205 downregulation correlated with both the mesenchymal phenotype and a more aggressive behavior	/
	Cell lines: H2452, MSTO-211H (Total, 2)	/	Cell line: SV40-transformed mesothelial cell line Met-5A (Total, 1)		NCodeTM miRNA qRT-PCR - Invitrogen (Ref Gene: RNU6B)	/		

**Nymark P *et al.* 2011 [70]**	13 lung tumors from asbestos-exposed patients (large cell lung cancer, adeno-squamous cell carcinoma, small cell lung cancer, adenocarcinoma)	/	8 control patients (hamartoma, lymph node, lymphoid hyperplasia, tuberculoma, inflammatory pseudotumor, lung abscess) and 13 lung tumors from non-exposed patients (large cell lung cancer, adeno-squamous cell carcinoma, small cell lung cancer, adenocarcinoma)	/	/	Agilent Human miRNA v2 G2565CA - Agilent Technologies	13 novel asbestos-related miRNAs were identified	Accession number: GSE25508
**Kemp CD *et al.* 2012 [93]**	Cell lines: H28, H2052, H2452, NCI-SB-MES1-4 (MPM primary culture) (Total, 4)	/	Cell lines: LP3, LP9, NCI-SB-NMES1-2 (isolated from normal pleura) (Total, 3)	Association of EZH2 with miRNA expression	qRT-PCR - Applied Biosystems (Ref Gene: RNU44)	/	EZH2 overexpression coincided with decreased miR-101 and miR-26a levels	/
	MPM tissue (n.a.)	/	Normal pleura tissue (n.a.)		(n.a.)	/		
**Weber DG *et al.* 2012 [88]**	Cellular fraction of peripheral blood: MM patients (Total, 23: 12 epith, 1 sarc, 7 biph, 3 other)	/	Cellular fraction of periferal blood: asbestos-exposed subjects (Total, 17) and healthy subjects (Total, 25)	Screening and validation	TaqMan MicroRNA assays - Applied Biosystem (Ref Gene: miR-125a)	MirVana miRNA Probe Set v2.0 - Ambion	34 down-regulated and 15 up-regulated miRNAs. A cut-off for miR-103 was determined to discriminate MM patients from asbestos-exposed and healthy subjects	Accession number: GSE29707 http://www.ncbi.nlm.nih.gov/geo/
**Tomasetti M *et al.* 2012 [86]**	Serum: MM patients (Total, 45 other)	/	Serum: NSCLC patients (Total, 20) and healthy subjects (Total, 56)	Clinical validation	qRT-PCR - Applied Biosystems (Ref Gene: U6 RNA, cel-miR-39)	/	MiR-126 differentiated MM patients from healthy controls and NSCLC from MM, but not NSCLC from control subjects	/
**Kirschner MB *et al.* 2012 [90]**	Plasma: MM patients (Total, 5: 3 epith, 2 sarc)	/	Plasma: healthy subjects (Total, 3)	Screening	/	Human 8x15K miRNA Microarray Kit V3 - Agilent Technologies	15 miRNAs showed significantly higher plasma levels in patients *vs.* controls; only 3 miRNAs had previously been reported	/
	Plasma: MM patients (Total, 15: 9 epith, 2 sarc, 3 biph, 1 other)	Free hemoglobin level < 0.25	Plasma: healthy subjects (Total, 14)	Test	microRNA-specific TaqMan Assays - Applied Biosistems (Ref Gene: miR-16)	/	MiR-625-3p, miR-29c*, and miR-92a levels were higher in MM patients *vs.* controls	
	Serum: MM patients (Total, 30: 29 epith, 1 other)	Free hemoglobin level < 0.25	Serum: asbestosis (Total, 10)	Validation	microRNA-specific TaqMan Assays - Applied Biosistems (Ref Gene: miR-16)	OpenArray platform - Applied Biosystens	MiR-625-3p levels discriminated MM patients from asbestosis patients (accuracy 79.3%, sensitivity 70%, specificity 90%)	
	FFPE tissue-LCM: MM (Total, 18: 15 epith, 3 biph)	LCM	FFPE tissue: pericardium (Total, 7)	Tissues analysis	microRNA-specific TaqMan Assays - Applied Biosistems (Ref Gene: RNU6B)	/	Only high miR-625-3p level was confirmed	
**Andersen M *et al.* 2012 [83]**	FFPE tissue: MPM (Total, 13 epith)	/	FFPE tissue: diagnostic biopsies (Total, 12) and NNP treat (Total, 13)	Analysis of 4 previously reported miRNAs	TaqMan MicroRNA Assays - Applied Biosystems (Ref Gene: RNU6B)	/	Significant downregulation of miR-17-5p and upregulation of miR-221 (opposite of expression pattern previously reported in cell lines)	/

**Xu Y *et al.* 2013 [75]**	Fresh frozen tissue: (Total, 25: 18 epith, 3 sarc, 4 biph)	> 80%	Fresh tissue: normal pleura (Total, 6)	Screening and validation	ViiA™ 7 RT-PCR System - Applied Biosystems (Ref Gene: RNU44)	Human miRNA BeadChip v2 - Illumina	MiR-1 is downregulated in MM and its forced expression induces growth arrest and apoptosis	Accession number: GSE40345 http://www.ncbi.nlm.nih.gov/geo/
**Reid G *et al.* 2013 [94]**	FFPE tissue: MPM (Total, 60: 46 epith, 16 biph)	/	FFPE tissue: normal pleura (Total, 23)	Analysis and validation of 4 miRNAs	microRNA-specific TaqMan assays - Applied Biosistems (Ref Gene: RNU6B)	/	MiR-15/16 family is downregulated and has tumor suppressor function in MPM	/
	Cell lines: H2052, H2452, H28, H226, MSTO-211H (Total, 5) and cell cultures: MM05 (primary mesothelioma cell line)	/	Cell line: MeT-5A (immortalized mesothelial cell line) (Total, 1)					
**Cioce M *et al.* 2013 [76]**	FFPE tissue: MPM (Total, 29: 25 epith, 3 biph, 1 desm)	/	FFPE tissue: mesothelial cysts (Total, 12)	Screening	/	Human miRNA microarray V2 Rel.16 - Agilent Technologies	MiR-145 downregulation is a characteristic trait of malignant *vs.* benign mesothelial tissue	/
	Fresh tissue: MPM (Total, 6), fresh frozen tissue: MPM (Total, 36)	/	Fresh-frozen tissue: normal pleura (Total, 14) and matched peritonea (Total, 36)	Double step validation	TaqMan MiRNA Assays - Applied Biosystems (Ref Gene: RNU6B, RNU49)	/		
	Cell lines: H2052, H2452, H28, H226, MSTO-211H (Total, 5) and cell cultures: MM05 (primary mesothelioma cell line)		Cell line (mesothelial cell line, Total, 1)					
**Cheng YY *et al.* 2013 [95]**	Cell lines: H2052, H2452, H28, H226, MSTO, REN (Total, 6) and cell cultures: MM05 (primary mesothelioma cell line)	/	Cell cultures: HMCs (normal human mesothelial cells) (Total, 1) and cell line MeT-5A (immortalized mesothelial cell line) (Total, 1)	Screening and validation	miRNA-specific TaqMan assays - Life Technologies (Ref Gene:	Ncode Human miRNA Microarray V3 system - Life Technologies	4 miRNAs were higher in MPM cells with reduced *ZIC1* expression	/
	FFPE tissue-LCM: MPM (Total, 27/31: 27 epith, 4 biph)	LCM	/	Analysis of expression status and survival	miRNA-specific TaqMan assays - Life Technologies (Ref Gene: RNU6B)	/	Higher expression in tumors correlates with shorter survival	
**Andersen M *et al.* 2014 [77]**	FFPE tissue: preoperative MPM diagnostic biopsy and post-treatment samples (Total, 5+5 epith)	40-85%	FFPE tissues: NNP post treatment (Total, 5)	Screening	miRCURY LNA Universal RT miRNA Ready-to-Use PCR, Human panels I + II version 2.M - Exiqon A/S (Ref Gene: SNORD49A)	/	23 miRNAs are differentially expressed between groups; chemotherapy reduces the differential expression	Accession number: GSE54394 http://www.ncbi.nlm.nih.gov/geo/
	FFPE tissues: post-treatment MPM samples (Total, 40: 18 epith, 22 biph)	>50%	FFPE tissues: NNP treat (Total, 14) and PTHX (Total, 5)	Double step validation	TaqMan miRNA Assays - Life Technologies (Ref Gene: SNORD49A)	/	A 4-miRNA (miR-126, miR-143, miR-145, miR-652, downregulated) diagnostic classifier distinguishes MPM from NNP with high overall	
	FFPE tissues: MPM preoperative diagnostic	>40%						

	biopsies (Total, 12: 9 epith, 3 biph)						accuracy	
**Riqueleme E *et al.* 2014 [96]**	Cell lines: H28, H2452, HP10, HP7, HCT4012, H2052, MSTO-211H (Total, 7)	/	/	MiRNA analysis of in 8q24 region	TaqMan MicroRNA Assays - Applied Biosystems (Ref Gene: U6)	/	Different expression level of miRNAs spanning the *PVT1* region in MPM cell lines	/
**Ramírez-Salazar EG *et al.* 2014 [78]**	FFPE macro-dissected MPM tissues: (Total, 5 epith)	> 80%	FFPE tissues: PP (Total, 4), HP (Total, 5), Ctrl group (adjacent non-cancerous - non-inflammatory tissue, Total, 5).	Profiling	/	TaqMan Array Human MicroRNA Panel v2.0 (A+B) - Applied Biosystems	Bioinformatic and network analysis of deregulated miRNAs (19 in MM *vs.* Ctrl group)	/
**Matsumoto S *et al.*2014 [97]**	FFPE macro-dissected MPM tissues: (Total, 25: 16 epith, 5 sarc, 4 biph)	> 90%	FFPE tissue: RMPs (Total, 20)	Analysis of miR-31	TaqMan Universal PCR Master Mix, No AmpErase UNG and TacMa miRNA-specific Primers - Applied Biosystems (Ref Gene: RNU6B)	/	MiR-31 is down-regulated in MM *vs.* RMP, but miR-31 upregulation in MM patients with sarcomatoid component is significantly associated with a worse prognosis	/
**Weber DG *et al.* 2014 [89]**	Cellular fraction of peripheral blood: MM patients (Total, 43: 28 epith, 5 sarc, 6 biph, 4 other)	/	Cellular fraction of periferal blood: asbestos-exposed subjects (Total, 52)	Analysis of miR-103a-3p	TaqMan miRNA Assays - Life Technologies (Ref Gene: miR-125a)	/	Combination of miR-103a-3p and mesothelin showed 95% sensitivity and 81% specificity in discriminating epith and biph MM from controls	/
**Kirschner MB *et al.* 2015 [79]**	FFPE tissues: 8 long-term and 8 short-term MPM survivors (Total, 16 epith)	LCM	8 epith short-term *vs.* 8 epith long-term survivors	Discovery set	/	Agilent Technologies Human 8x15 k microRNA Array Kit V3 - Agilent Technologies	A 6-miRNA signature (miR-score) is associated with longer survival in MM patients undergoing EPP and palliative surgery	Accession number: GSE59180 http://www.ncbi.nlm.nih.gov/geo/
	FFPE tissues: MPM EPP including patients who received chemotherapy (13/48) and those who died <8 weeks after surgery (2/48) (Total, 48: 31 epith, 17 biph)	LCM	Median expression of miRNAs observed across all samples	Training set	Stem-loop primers and hydrolysis probes - Life Technologies (Ref Gene: RNU6B)	/		
	FFPE tissue: MPM patients undergoing (Total, 43: 25 epith, 5 sarc, 13 biph)	LCM		Validation set				
**Ak G *et al.* 2015 [80]**	Fresh-frozen tissue: MPM patients (Total, 18: 10 epith, 4 sarc, 4 biph)	> 50%	Fresh-frozen tissue: from BAPE patients (Total, 6)	Profiling	/	TaqMan Human MicroRNA Array Card A - Applied Biosystems	11 significantly up-regulated miRNAs in MPM compared with BAPE	/
**Birnie KA *et al.* 2015 [98]**	Cell lines: NO36, JU77, LO68 (MPM cell lines) (Total, 3)	/	Primary human mesothelial cells and Met-5A	Profiling and Validation of miR-223	TaqMan Universal PCR Master Mix, No AmpErase UNG - Life Technologies (Ref Gene: RNU48, RNU44, SNOR202)	Taqman Open Array - Life Technologies	MiR-223 showed reduced levels in MPM specimens. MiR-223 targets STMN1, a microtubule regulator associated with MPM.	/

	Cell lines: NO36, JU77, LO68, CRL2081, CRL5820 (MPM cell lines) (Total, 5) and mouse MPM cel lines (Total, 3)	/	Human and mouse mesothelial cells	Validation of miR-223	TaqMan Universal PCR Master Mix, No AmpErase UNG - Life Technologies (Ref Gene: RNU48, RNU44, SNOR203)	/		
	FFPE tissues: MPM (Total, 17: histology n.a.)	/	FFPE tissues: pericardial mesothelium samples (Total, 6)	Validatioin of miR-223	TaqMan Universal PCR Master Mix, No AmpErase UNG - Life Technologies (Ref Gene: RNU6B)	/		
	Cells from pleural effusion of MPM patients (histology n.a.)	/	Cells from pleural effusion of benign diseases	Profiling and Validation of miR-223	TaqMan Universal PCR Master Mix, No AmpErase UNG - Life Technologies (Ref Gene: RNU6B)	Taqman Open Array - Life Technologies		
**Williams** ***M et al.*** **2015 [99]**	FFPE tissues: 59 EEP and 61 P/D (Total, 120: 72 epith, 9 sarc, 39 biph)	/	FFPE tissue: normal mesothelium (Total, 23)		See Reid G et al. 2013 and Kirschner MB et al. 2014	/	Levels of mature miR-193a-3p and miR-192 are reduced in MPM	/
	Cell lines H28, H2052, H2452, H226, MSTO-211H, MM05, VMC23, P31 , SPC111 and SPC212 (Total, 10)	/	MeT-5A, non-small cell lung cancer line A549 and LP9 (Total, 3)		TaqMan low-density array cards - LifeTechnologies (Ref Gene: RNU48)	/		
**Lamberti M** ***et al.*** **2015 [91]**	Serum: MM patients (Total, 14: 7 epith, 3 sarc, 4 biph)	/	Serum: non-cancer-related pleural effusion patients (Total, 10)	Profiling and Validation	TaqMan microRNA Assays - Applied biosistems (Ref Gene: miR-16)	Megaplex Microfluidic Card Pool A - Applied Biosystems	Two distinctive miRNA signatures predicting histotype and survival in patients	

Note: characteristics of samples, miRNA detection methods, relevant findings, submission of array data to a public repository (GEO or ArrayExpress) are reported. Studies are listed by year of publication. The following abbreviations describe sample characteristics as reported in relevant papers. **BAPE:** benign asbestos-related pleural effusion, **biph:** epithelioid-sarcomatoid, **Ctrl group:** adjacent non-cancerous – non-inflammatory tissue, **DB:** preoperative diagnostic biopsies, **EPP:** extrapleural pneumonectomy, **epith:** epithelioid, **HP:** atypical mesothelial hyperplasia, **lym:** lymphohistiocytic, non-epithelioid or sarcomatoid and mixed, **n.a.:** not available, **NNP:** patient-matched non-neoplastic pleura, **PB:** peripheral blood, **P/D:** pleurectomy ± decortication, **PP:** pachypleuritis/chronic inflammation, **PTHX:** non-neoplastic reactive mesothelial proliferation due to pneumothorax, **RMPs:** reactive mesothelial proliferations, **sarc:** sarcomatoid, **treat:** three series of cisplatin and vinorelbine treatments, **WT:** non-transfected MM cell line.
